# Experimental and Theoretical Investigation of Diffusion Characteristics of CH_4_ and CO_2_ in Shales

**DOI:** 10.3390/nano16181178

**Published:** 2026-09-17

**Authors:** Jingjing Guo, Minjie Mao, Chaozhi Jiang

**Affiliations:** 1 State Key Laboratory of Oil and Gas Reservoir Geology and Exploitation, Southwest Petroleum University, Chengdu 610500, China; 2 Shunan Gas Mine of PetroChina Southwest Oil and Gas Field Company, Luzhou 646001, China

**Keywords:** gas shales, CH_4_/CO_2_ diffusion, apparent diffusion coefficient, influential factors

## Abstract

The diffusion characteristics of CH_4_ and CO_2_ in micro–nanopores of shale gas reservoirs exert a significant impact on the production performance of supercritical CO_2_-enhanced shale gas recovery. High-temperature and high-pressure experimental investigations on shale gas diffusion are still relatively limited compared with abundant laboratory studies under conventional conditions. To investigate the diffusion kinetic behavior of CH_4_ and CO_2_ in shale micro–nanopores under high-temperature and high-pressure conditions, a series of isobaric diffusion experiments across variable pressure and temperature conditions were performed on marine shale samples from the Longmaxi Formation in the Sichuan Basin, China. A diffusion coefficient model that incorporates both bulk diffusion and surface diffusion mechanisms was adopted to fit the experimental data, and the influences of shale pore size, temperature, and pressure on the diffusion behaviors of CH_4_ and CO_2_ in shale reservoirs were systematically analyzed. The results indicate the following: (1) Under identical high-temperature and high-pressure conditions, the diffusion coefficient of CH_4_ in shale nanopores is larger than that of CO_2_. (2) The diffusion coefficients of CH_4_ and CO_2_ exhibit a positive correlation with temperature and a negative correlation with pressure. (3) Under the coupled influence of temperature and pressure, the diffusion coefficients of CH_4_ and CO_2_ generally exhibit a decreasing trend as both temperature and pressure increase simultaneously. (4) The pore size distribution of shale samples has a significant effect on the diffusion characteristics of both gases. The larger the overall pore size of the shale, the greater the diffusion coefficients of CH_4_ and CO_2_ under identical temperature and pressure conditions. The research findings provide valuable insights for the dynamic prediction of supercritical CO_2_ enhanced shale gas recovery.

## 1. Introduction

Unconventional shale gas has become a key pillar for natural gas production growth in China, and is of great significance for achieving the national “dual-carbon” strategic objectives. The combined effects of multiple occurrence states of shale gas and the extremely low permeability of reservoirs leads to significant differences in gas migration characteristics between shale reservoirs and conventional gas reservoirs, where gas flow in shales is governed by the integrated action of multiple mechanisms including desorption, diffusion and viscous flow. Among these mechanisms, diffusion is a particularly critical mechanism for gas migration within shale reservoirs. An accurate understanding of the gas diffusion behaviors in shale reservoirs is critical for unraveling gas migration laws and optimizing productivity prediction models. Furthermore, injecting supercritical CO_2_ into shale gas reservoirs can effectively increase gas production and enhance shale gas recovery; however, the diffusion behaviors of CO_2_ within shale nanopores under in situ conditions still require in-depth investigation.

Research on the diffusion behaviors of hydrocarbon gases in nanopores can divided into two major categories: experimental characterization and theoretical modeling [1]. Javadpour et al. [2,3] proposed the concept of apparent permeability based on experimental measurements, which enables a continuous quantitative description of slip flow and transition flow regimes. Du et al. [4] conducted adsorption kinetics experiments on marine (Wufeng Formation) and continental (Yanchang Formation) shale samples via the volumetric method, under temperatures of 318 K, 338 K, and 358 K and pressures ranging from 3.0 MPa to 8.0 MPa, to obtain the diffusion coefficients of CH_4_ and CO_2_. Zou [5] conducted experiments on five shale samples collected from the Perth Basin in Western Australia to obtain CH_4_ adsorption volume–time curves, and further fitted the curves using a dual-pore diffusion model to analyze the effects of temperature and pressure on diffusion coefficients. In general, the number of experimental studies focusing on shale gas diffusion under high-temperature and high-pressure conditions remains relatively limited. Zou et al. [6] conducted CH_4_ diffusion experiments on shale core samples at temperatures up to 150 °C and pressures up to 35 MPa, and found that the diffusion coefficient of CH_4_ in shale decreases with increasing pressure and increases with rising temperature. This work also implemented regression fitting on the experimental results via a theoretical diffusion coefficient model, and on this basis, quantitatively analyzed the influences of temperature, pressure, pore size and other factors on the total diffusion coefficient of CH_4_. Nevertheless, the maximum experimental pressure adopted in this investigation still remains relatively low, and the study only focuses on diffusion behaviors of CH_4_ in shales, without extending to other gas components. Numerous high-pressure adsorption–desorption experiments have demonstrated that CO_2_ exhibits higher adsorption capacity and stronger competitive adsorption selectivity than CH_4_ in shales. Meanwhile, temperature–pressure conditions, mineral composition, and organic matter content significantly affect gas adsorption capacity and diffusion-transport behavior in shales [7,8,9,10,11].

Considering the limitations of experimental temperature and pressure conditions, some researchers have adopted theoretical models and molecular simulation methods to analyze gas diffusion characteristics under in situ reservoir conditions. Based on Hwang’s theoretical framework, Wu et al. [12] introduced a correction term accounting for high-pressure adsorption layer coverage, and further developed a surface diffusion model incorporating surface energy heterogeneity, adsorption heat gradient and non-isothermal desorption effects. Li et al. [13] combined grand canonical Monte Carlo and molecular dynamics (GCMC-MD) simulation methods to conduct numerical simulation studies on the adsorption and diffusion characteristics of shale gas in nanopores. Zhou et al. [14] constructed nanopore models of shale organic matter (kerogen) and inorganic matter (kaolinite), and analyzed the diffusion behaviors of CH_4_/CO_2_ under varying temperatures, pressures and pore sizes using grand canonical Monte Carlo (GCMC) and molecular dynamics (MD) simulations. Hamza [15] evaluated the impact of clays on CO_2_ adsorption for different sandstone samples with varying clay contents and types, covering a temperature range of 50–100 °C and pressures up to 20 MPa. Jin [16] employed the dual control volume-grand canonical molecular dynamics (DCV-GCMD) simulation method to investigate the flow and diffusion mechanisms of methane in 1–4 nm shale nanopores under varying temperature and pressure conditions. Theoretical approaches including molecular dynamics and GCMC simulations can microscopically resolve the competitive adsorption, diffusion and displacement processes of CO_2_ and CH_4_ in shale nanopores, and clarify the modulation mechanisms of pore structure and environmental conditions on molecular transport behaviors [17,18,19,20,21,22,23,24].

Deep shale gas reservoirs in the Sichuan Basin of China are generally characterized by reservoir pressures exceeding 60 MPa and temperatures above 80 °C [25,26,27]. Investigations on gas diffusion behaviors in shales under high-temperature and high-pressure conditions can provide fundamental support for the efficient exploitation of deep shale gas resources. Taking shale core samples from the Longmaxi Formation in a deep shale gas block of the Sichuan Basin as the research object, the isobaric diffusion method was adopted to measure the diffusion coefficients of supercritical CO_2_ and CH_4_ under varying pressures and temperatures, and the discrepancies in the diffusion performance of CO_2_ and CH_4_ in shales under different temperature and pressure conditions were quantitatively revealed. Combined with theoretical calculation of diffusion coefficients, this study further elucidates the diffusion characteristics of different types of gases under the influences of temperature, pressure and pore radius.

## 2. Experimental Determination of Diffusion Coefficients

### 2.1. Experimental Samples

The shale core sample used in this study was obtained from the Lower Silurian Longmaxi Formation in southern Sichuan Basin, China. The sampling interval belongs to typical marine shale sedimentary strata with stable burial depth and complete stratigraphic preservation, which is a major shale gas producing formation in China. The sampling depth is approximately 3700 m, which is representative of the prime production zone in this play. The reservoir temperature ranges from 383.15 K to 393.15 K, and the original formation pressure is about 75 MPa. Constrained by the field coring schedule and on-site engineering constraints, only one representative shale core sample from the Longmaxi Formation was collected for the diffusion experiment in this study. Further systematic investigation involving multiple core samples from the same target block will be conducted in follow-up work to improve the generality and robustness of the presented conclusions. The basic parameters of the core samples are presented in Table 1.

X-ray diffraction (XRD) and total organic carbon (TOC) tests were conducted on the collected shale core sample to determine its mineral composition and total organic carbon content. The corresponding test results are summarized in Table 2 and Table 3. The main mineral components (in wt%) of the sample are as follows: quartz 67.7%, plagioclase 3.3%, calcite 14.2%, dolomite 9.6%, pyrite 0.8%, and clay minerals 4.4%.

The ideal chemical formulas corresponding to the main mineral components of the core sample are SiO_2_, Na[AlSi_3_O_8_] (albite)/Ca[Al_2_Si_2_O_8_] (anorthite), CaCO_3_, CaMg(CO_3_)_2_, FeS_2_, K_0.65_Al_2_[Al_0.65_Si_3.35_O_10_](OH)_2_, and (Mg,Fe,Al)_3_(Si,Al)_4_O_10_(OH)_2_·(Mg,Fe,Al)_3_(OH)_6_, respectively. The standard verified crystallographic parameters of all identified main mineral phases (quartz, plagioclase, calcite, dolomite, pyrite, illite, chlorite, and illite–smectite mixed layer), which are retrieved from the American Mineralogist Crystal Structure Database, are summarized in Table 4.

**Figure 1:**
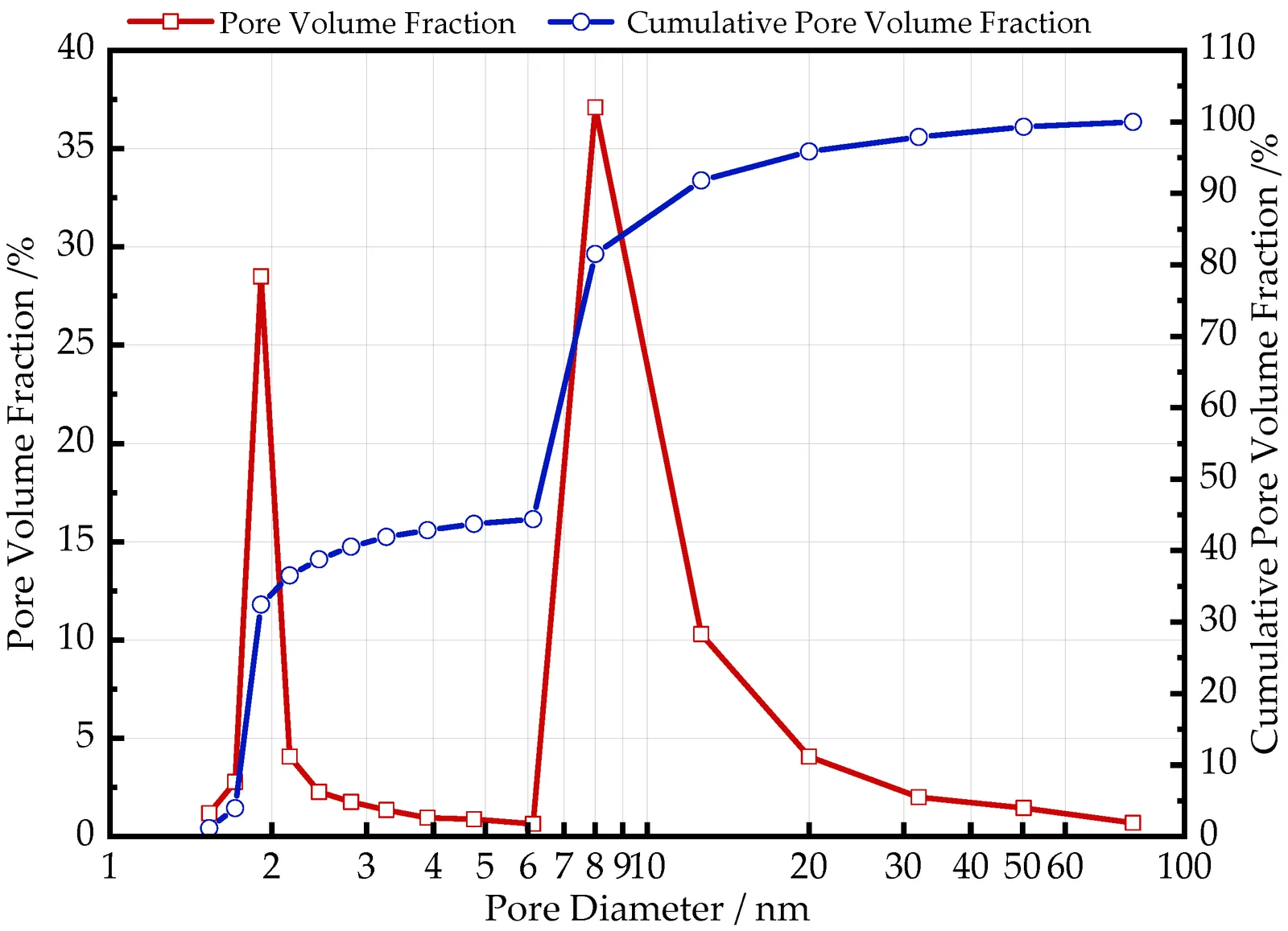
Pore size distribution of the shale sample obtained by combining MIP and LTNA.

Mercury intrusion porosimetry (MIP) combined with low-temperature nitrogen adsorption (LTNA) was employed to characterize the full-scaled pore size distribution characteristics of the core sample, and the results are presented in Figure 1. The results show that the pore diameter distribution curve of the core sample exhibits typical bimodal distribution characteristics, indicating the development of multi-scale pore spaces in the core sample. The average pore diameter of the core sample is 7.98 nm, among which micropores (<2 nm) and mesopores (2–50 nm) account for 37.26% and 62.02%, respectively. It is evident that large amounts of nanopores are developed in the core sample.

It should be noted that shale exhibits strong heterogeneity, which exerts a significant effect on gas diffusion behavior. Specifically, the mineral composition determines the surface roughness and pore wall properties of shale pores: higher contents of clay and quartz tend to complicate the pore structure and correspondingly increase gas diffusion resistance. The organic matter content provides abundant micropore spaces for absorbed gas storage and diffusion, and its pore development degree directly governs the diffusion velocities of CH_4_ and CO_2_ in shale matrices.

### 2.2. Experimental Principle

The isobaric diffusion method was adopted to determine the diffusion coefficients of CH_4_ and CO_2_ in the shale core sample. The isobaric diffusion equation is obtained based on two core assumptions: (1) The gas in both the upstream and downstream chambers remains fully mixed at all times, with no concentration gradient inside the chambers. (2) The shale core sample is the only mass transfer barrier between the two chambers, and gas leakage at the core holder sealing interface is negligible. These assumptions are consistent with the standard isobaric diffusion test specifications specified in SY/T 6129-2016 [28].

The inlet and outlet surfaces of the shale core sample are set as constant-concentration boundaries, which are determined by the real-time gas concentration measured by the high-precision concentration sensors installed in both chambers. No-slip and no-mass-loss constraints are applied on the radial circumferential surface of the core, which is in close contact with the sealed core holder.

Based on the mass conservation equation and Fick’s second law of diffusion, the gas diffusion coefficient in porous core media can be calculated as follows:

(1) $$D_{F}=-L\ln\frac{C_{t1}-C_{t2}}{C_{01}-C_{02}}/(St\left(\frac{1}{V_{1}}+\frac{1}{V_{2}}\right))$$

If we define $K=\frac{S}{L}\left(\frac{1}{V_{1}}+\frac{1}{V_{2}}\right)$, then Equation (1) can be rewritten as follows:

(2) $$\ln\left(\frac{C_{t1}-C_{t2}}{C_{01}-C_{02}}\right)=-KD_{F}t$$

where *D*_F_ is the measured gas diffusion coefficient, cm^2^/s; (*C*_01_ − *C*_02_) is the initial CH_4_ or CO_2_ concentration difference between the two diffusion chambers A and B, %; (*C*_t1_ − *C*_t2_) is the CH_4_ or CO_2_ concentration difference between the diffusion chambers A and B at a given time, %; *S* is the cross-sectional area of the tested core sample, cm^2^; *L* is the length of the tested core sample, cm; *V*_1_ and *V*_2_ are the volumes of chamber A and chamber B, cm^3^; and *t* is the diffusion time, s.

The volumes of both upstream and downstream chambers (i.e., *V*_1_ and *V*_2_) have been accurately calibrated using the helium expansion method before the formal test. During the entire diffusion process, we maintain a constant temperature and pressure environment, so the chamber volumes are treated as fixed.

It can be observed that the term ln(*C*_t1_ − *C*_t2_/*C*_01_ − *C*_02_) exhibits a linear relationship with diffusion time *t*, with a slope of *m* = −*KD*_F_. By fitting the slope *m* of the ln(*C*_t1_ − *C*_t2_/*C*_01_ − *C*_02_)~*t* linear regression curve based on experimental data, the diffusion coefficient can thus be calculated as follows:

(3) $$D_{F}=\left(\ln\frac{C_{01}-C_{02}}{C_{t1}-C_{t2}}\right)/\left[\frac{S}{L}\left(\frac{1}{V_{1}}+\frac{1}{V_{2}}\right)t\right]$$

### 2.3. Experimental Apparatus and Procedures

Shale gas reservoirs are characterized by extensively developed nanoscale pore networks, and the diffusion behaviors of CO_2_ and CH_4_ within nanopores are influenced by multiple factors including formation temperature, formation pressure, and pore diameter. Among these factors, pressure and temperature are identified as two primary factors affecting gas diffusion kinetics [3,4,5,29,30]. To systematically clarify the diffusion characteristics of CH_4_ and CO_2_ under the high-temperature and high-pressure conditions, a series of diffusion coefficient measurement experiments for both CH_4_ and CO_2_ were conducted on the typical shale sample from the Longmaxi Formation. The experiments were performed under different temperatures (323.15 K, 343.15 K, 363.15 K, 383.15 K), with the diffusion chamber pressure ranging from 20 MPa to 50 MPa and the confining pressure set between 30 MPa and 60 MPa. It should be noted that, constrained by the maximum safe operating pressure of the experimental apparatus, the pore pressure set in this study ranges from 20 MPa to 50 MPa. This pressure interval fully covers the main pressure range during middle-late production stage of the target deep shale gas reservoirs in the Sichuan Basin, and the experimental results can thus reliably represent the gas diffusion behaviors under actual field production conditions.

The experiments conducted in this work are consistent with the actual geological parameters of the target marine shale gas reservoirs in the Sichuan Basin in China. The target shale layer is dominated by methane (usually accounting for more than 95% of total shale gas components), with only trace amounts of ethane and other heavy hydrocarbon components. No large-scale free formation water exists in the reservoir, and only an immobile bound water film is adsorbed on the surface of hydrophilic nanopores. The bound water film will not participate in macroscopic flow or cause convective interference to gas diffusion, and only slightly reduces the effective diffusion cross-section of local pores. Therefore, there is no two-phase flow of gas and water in the entire experimental and simulation system, and only single-phase gas flow occurs. Therefore, in the diffusion experiments, pure methane was adopted as the medium gas, corresponding to the dominant gas component in the in situ reservoir. Furthermore, in order to provide guidance for evaluating the performance of underground gas storage (UGS) in depleted shale gas reservoirs, pure CO_2_ was also adopted as the medium gas to analyze its diffusion behaviors.

The self-developed QTKS-2 gas diffusion coefficient measurement apparatus was used to determine the diffusion coefficients of CH_4_ and CO_2_ in shale samples under different pressures and temperatures. The main components of the experimental setup include diffusion chambers, a temperature control system, high-accuracy pressure sensors, a high-pressure gas injection manifold, a gas chromatograph for component concentration detection, and a real-time data acquisition system. Given the strong corrosiveness of supercritical CO_2_, corrosion-resistant fluorine-containing rubber sealing rings were selected for all experiments to ensure the sealing integrity of the high-pressure vessel during the whole experiment process. In this experimental setup, pore pressure and confining pressure are adjusted synchronously. The difference between confining pressure and pore pressure, i.e., the effective stress, is maintained at a constant value of 10 MPa across the entire pressure test sequence. This design completely eliminates the interference of variable effective stress on pore deformation and diffusion behavior.

In the isobaric diffusion experiment, N_2_ was selected as the inert reference gas strictly following the shale gas diffusion measurement specifications specified in the industry standard SY/T 6129-2016. Under the designed experimental conditions, N_2_ is assumed to be completely inert. Previous studies have verified that even under high-temperature and high-pressure conditions, N_2_ does not undergo decomposition or chemical reaction with the shale matrix, and its adsorption capacity on shale is far lower than that of CH_4_ and CO_2_, which can be almost ignored. Therefore, throughout the entire experiment process, N_2_ does not participate in the gas diffusion process in the shale core, nor does it alter the pore structure properties of the shale matrix or the gas-phase transport properties.

The role of nitrogen (N_2_) in the diffusion experiment can be classified into two categories. First, high-purity N_2_ was used for vacuum purging and pretreatment of shale samples prior to the diffusion coefficient measurement, to remove residual air, moisture and impurity gases in the shale pore throats and experimental pipeline, and eliminate potential interference of impurities on CH_4_ and CO_2_ diffusion experiments. Furthermore, high-purity nitrogen precisely maintains consistent pressure on both sides of the dual-chamber diffusion system, which effectively eliminate the seepage-driven mass transport interference, and ensures that the entire gas diffusion process is purely governed by the concentration gradient of the target gas.

Furthermore, the gas concentration detection system we adopted has been calibrated for mixed gas components, which can accurately distinguish the concentration signals of N_2_ and target diffusion gas (CH_4_ or CO_2_). Since N_2_ does not participate in the diffusion process inside the shale core sample, the mutual diffusion between CH_4_/CO_2_ and N_2_ in the free gas phase can be completely ignored. This ensures that the mass transport behavior in the porous shale medium is dominated by the single-component diffusion of CH_4_ or CO_2_, which is consistent with the standard technical requirements for shale gas diffusion coefficient measurement.

**Figure 2:**
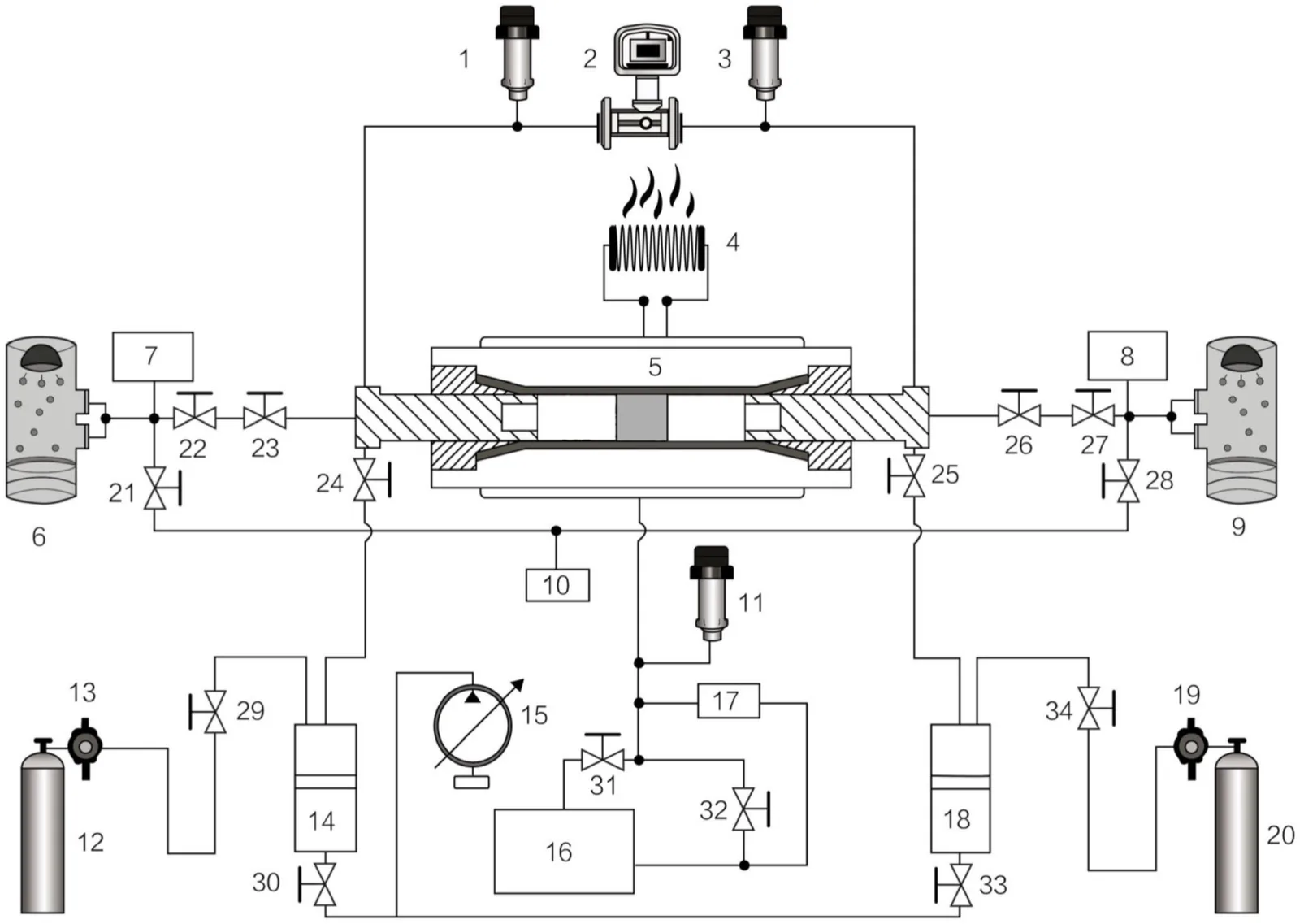
Schematic diagram of the experimental set-up for gas diffusion coefficient measurement in shales; 1, 3, 11—pressure sensors; 2—differential pressure sensor; 4—constant temperature oven; 5—core holder; 6, 9—gas chromatograph; 7, 8—relief valve; 10—vacuum pump; 12—gas source; 13, 19—multi-port valves; 14, 15, 18—gas booster; 16—confining pressure tracking pump; 17—safety valve; 20—gas source; 21–34—valves.

The detailed experimental procedure is as follows:

(1) Turn on the gas chromatograph to complete the pre-run calibration for on-line component detection, then load the shale core plug into the core holder, and connect all units of the experimental setup in accordance with the schematic diagram shown in Figure 2.

(2) Fully vacuum the entire experimental system and inject high-purity CH_4_ (or CO_2_) and N_2_ into the two intermediate containers, respectively.

(3) Heat the whole system to the predesigned temperature, and maintain the constant-temperature condition for 2 to 2.5 h to ensure a uniform temperature distribution across the entire setup.

(4) Stepwise increase the confining pressure and chamber pressure. Since both the target confining pressure and pore pressure exceed 10 MPa, a staged loading strategy is adopted to avoid mechanical damage or crushing of the shale sample. An initial confining pressure of no less than 3 MPa is applied first. After that, inject CH_4_ (or CO_2_) and high-purity N_2_ into the two separate diffusion chambers simultaneously to raise their pressure synchronously. Then, increase the confining pressure in small increments correspondingly, until both the preset target confining pressure and pore pressure are reached. During the entire gas injection process, the pressure difference between the two diffusion chambers is strictly controlled to keep their pressure fully consistent.

(5) After the gas charging procedure is completed, use the pre-calibrated gas chromatograph to analyze the gas composition and measure the concentration of CH_4_ (or CO_2_) in both diffusion chambers at fixed time intervals, and then calculate the corresponding diffusion coefficients of CH_4_ (or CO_2_) in the shale sample. Under our experimental conditions (323.14 K~383.15 K, 20 MPa~50 MPa), both pure CH_4_ and pure CO_2_ are consistently maintained in the supercritical state, as the temperature and pressure ranges far exceed the critical temperature and critical pressure thresholds of the two gases. This phase state is fully consistent with the actual in situ occurrence environment of deep marine shale gas reservoirs in the Sichuan Basin, China.

(6) Change the experimental pressure and temperature, and repeat steps 1 to 5 to obtain the apparent diffusion coefficients of CH_4_ (or CO_2_) in shales under various conditions.

It should be noted that all diffusion experiments under identical conditions were repeated at least three times. The final diffusion coefficient was determined from the averaged results of these parallel replicate tests.

## 3. Experimental Results and Discussion

### 3.1. Diffusion Behaviors of CH_4_ and CO_2_ Under Different Temperatures

**Figure 3:**
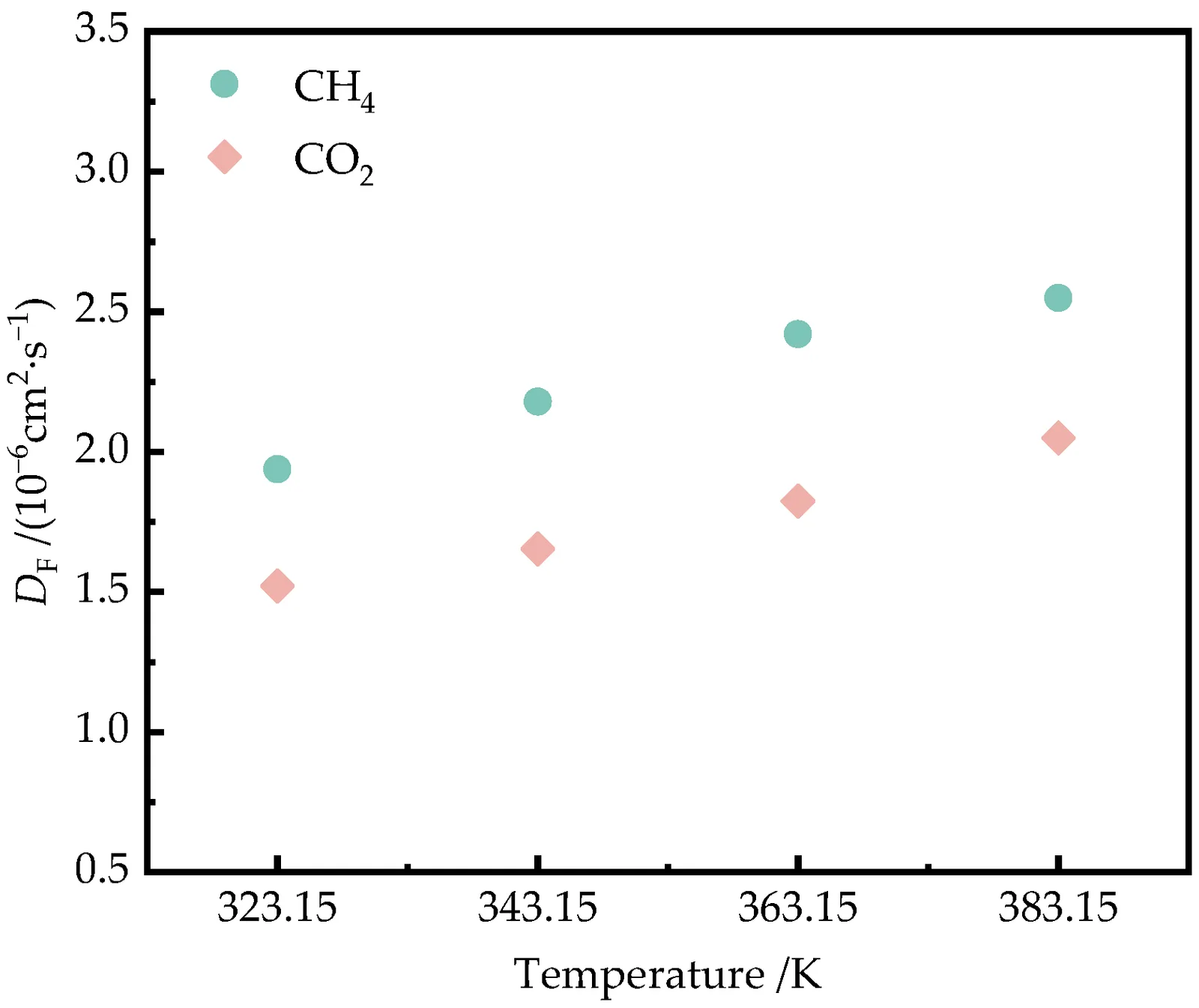
Diffusion coefficients of CH_4_ and CO_2_ as a function of temperature.

In order to quantitatively clarify the effect of temperature on gas diffusion behaviors in shale reservoirs, the diffusion coefficients of CH_4_ and CO_2_ in the typical shale core sample were measured under different temperatures, with the confining pressure maintained at 60 MPa and the diffusion chamber pressure kept constant at 50 MPa. The measured diffusion coefficients for both gases in the heterogeneous shale core sample at different temperatures are presented in Table 5 and Figure 3. The corresponding fitting curves of diffusion coefficients are provided in the Supplementary Materials (Online).

As illustrated in Figure 3, the diffusion coefficients of CH_4_ and CO_2_ in shale exhibit a positive correlation with temperature. As the experimental temperature rises from 323.15 K to 383.15 K, the diffusion coefficients of CH_4_ and CO_2_ increase by 31.53% and 34.71%, separately. This is because the thermal motion rate of gaseous molecules is significantly affected by temperature. An increase in temperature directly enhances the kinetic energy of gas molecules, thereby accelerating their diffusion efficiency within the shale matrix. This variation trend is consistent with previous studies in the literature [5,6,29]. Notably, under identical temperature and pressure conditions, the diffusion coefficient of CO_2_ in the tested shale sample is consistently lower than that of CH_4_ [30,31,32], which may be attributed to the predominance of Fick diffusion in the sample.

### 3.2. Diffusion Behaviors of CH_4_ and CO_2_ Under Different Pressures

**Figure 4:**
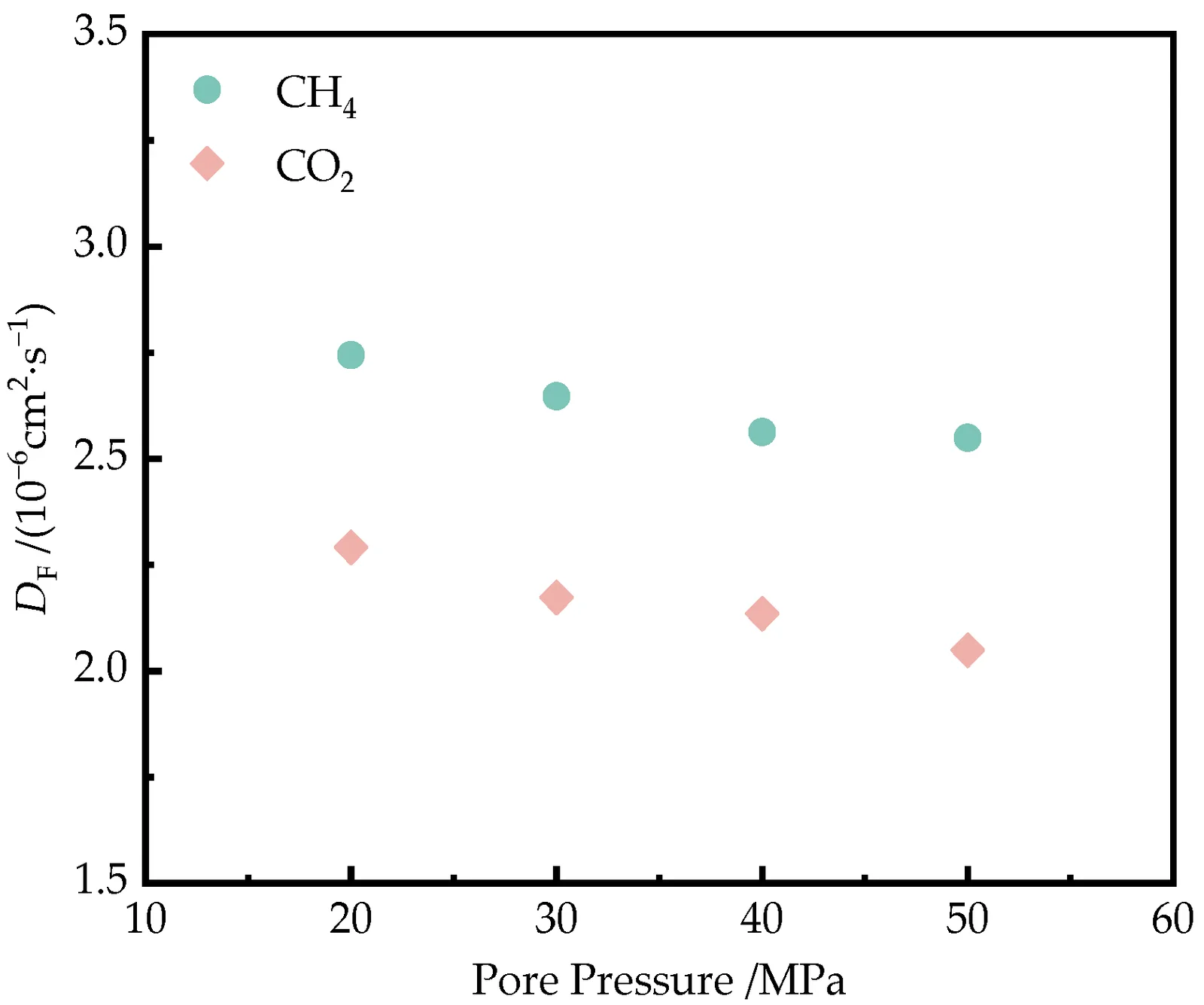
Diffusion coefficients of CH_4_ and CO_2_ as a function of pressure.

Table 6 and Figure 4 present the measured diffusion coefficients for CH_4_ and CO_2_ in the heterogeneous shale core sample under varying pressures at a constant temperature of 383.15 K. As shown in Figure 4, at a fixed temperature of 383.15 K, the diffusion coefficients of CH_4_ and CO_2_ in shale matrix decrease gradually with increasing pressure, and the rate of decline slows progressively as pressure rises. This variation trend aligns with prior studies: Rani et al. observed diffusion coefficients of CH_4_ and CO_2_ inversely proportional to pressure (2–8.9 MPa) based on adsorption kinetics experiments conducted on two shale samples from the Damodar Valley Basin, India [31], and Davarpanah and Mirshekari also verified that increasing pressure inhibited the diffusion of CH_4_ and CO_2_ in shales [32]. Our results under high-temperature and high-pressure conditions are in agreement with these earlier findings obtained under relatively lower pressure and temperature conditions.

## 4. Theoretical Diffusion Model of CH_4_ and CO_2_ in Shales

Gas diffusion in actual shale reservoirs results from the coupled effects of two primary mechanisms: bulk diffusion of free-state gas (Fick diffusion and Knudsen diffusion) and surface diffusion of adsorbed-state gas. The experimentally measured gas diffusion coefficients actually represent an apparent diffusion coefficient reflecting the combined effects of multiple diffusion mechanisms across different pore scales, and the individual contribution and governing patterns of each specific diffusion mechanism cannot be directly distinguished from the experimental data alone. To quantitatively analyze the contribution proportions of Fick diffusion, Knudsen diffusion and surface diffusion, and to identify the dominant controlling factors of gas diffusion in complex shale matrix, a mathematical model incorporating coupled multi-scale temperature and pressure conditions was employed in this study [33].

### 4.1. Bulk Gas Diffusion Coefficient

**Figure 5:**
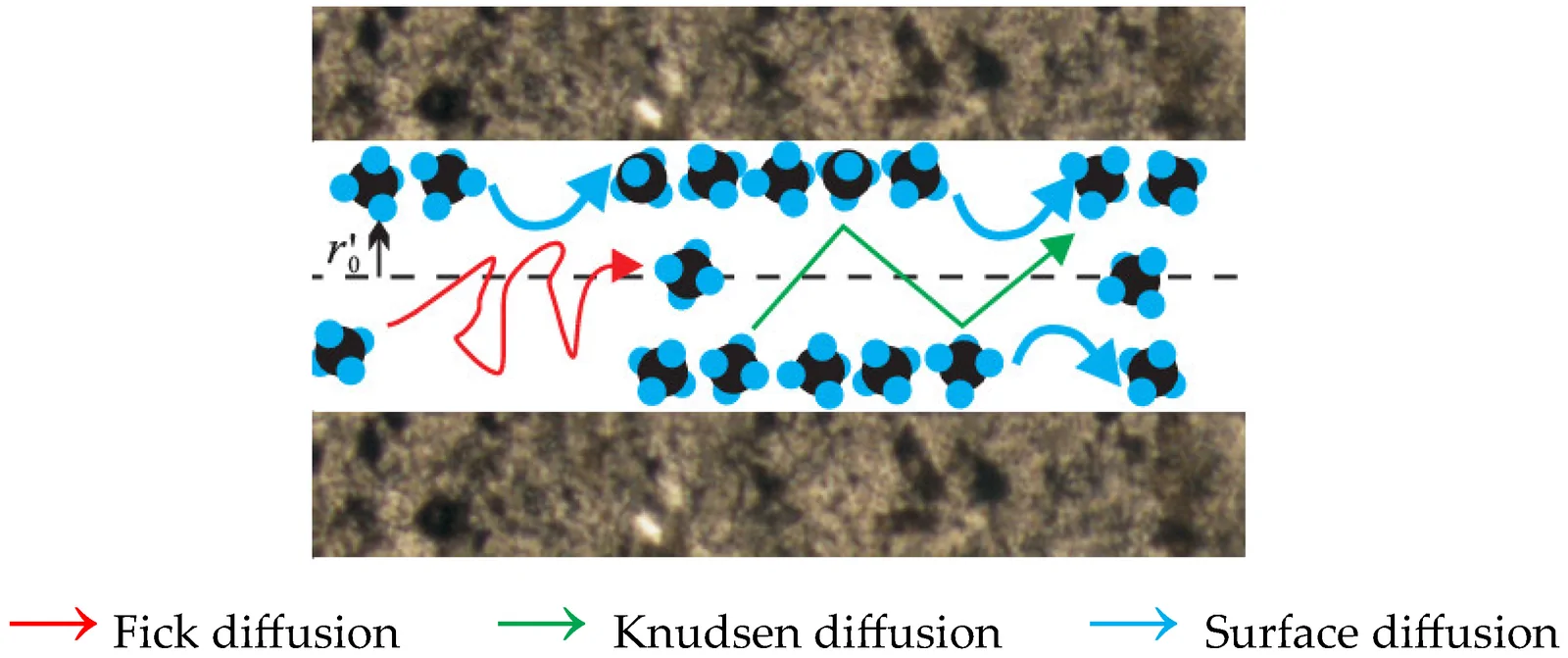
Schematic diagram of the bulk gas diffusion behaviors in shale matrix pores [6]. Fick diffusion dominates in relatively large pores, where the mass transport process governed by inter-molecule collisions. Knudsen diffusion prevails in smaller nanopores, governed by collisions between gas molecules and pore walls. The relative contribution of the two diffusion mechanisms varies with in situ pore size and pressure conditions.

Bulk gas diffusion in shale pores can be divided into Knudsen diffusion and Fick diffusion based on the Knudsen number (*Kn*), as shown in Figure 5 [6]. The Knudsen number is defined as the ratio of the mean free path of gas molecules to the pore diameter:

(4) $$Kn=\frac{\lambda }{d}$$

where *d* is the pore diameter, nm; *λ* is the mean free path of gas molecules, nm.

The mean free path of gas molecules can be determined by the following equation:

(5) $$\lambda =\frac{k_{b}T}{\sqrt{2}\pi \delta^{2}p}$$

where $k_{b}$ is the Boltzmann constant, with a value of 1.3805 × 10^−23^ J/K; *T* is the absolute temperature, K; δ is the collision diameter of gas molecules, m; *p* is the gas pressure, Pa.

When the Knudsen number (*Kn*) is less than or equal to 0.1, gas diffusion in porous media falls within the Fick diffusion regime. In this case, the mean free path of gas molecules is significantly smaller than the pore size, and the diffusion process is mainly dominated by inter-molecular collision. This type of gas diffusion typically occurs in large pores or under high-pressure conditions, where frequent inter-molecular collisions give rise to a relatively high diffusion rate. In contrast, when *Kn* > 0.1, gas diffusion in porous media falls into the Knudsen diffusion regime. This type of diffusion predominantly occurs in low-pressure environments or within nanoscale pores, where gas molecules collide more frequently with the pore walls rather than with each other. This wall-collision-dominated diffusion mode results in a reduced overall diffusion rate [33,34].

**Figure 6:**
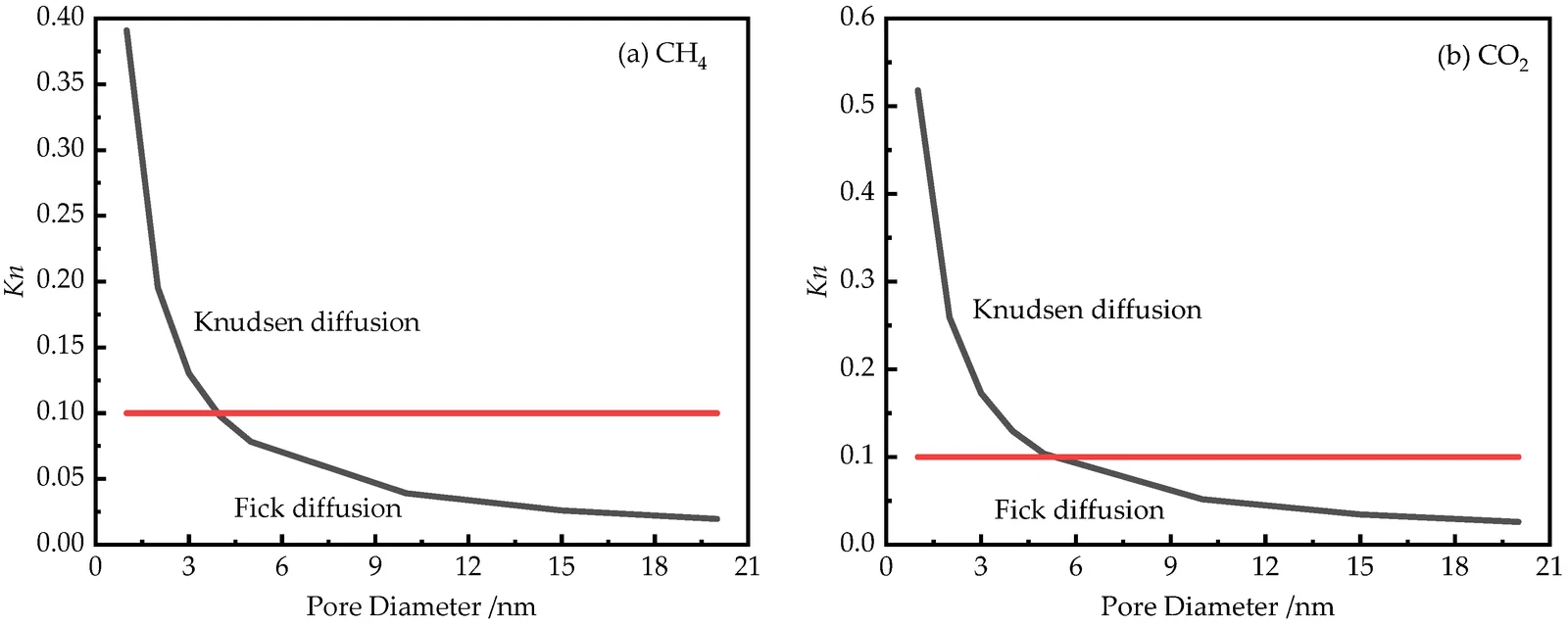
Curves of Knudsen number (*Kn*) versus pore diameter in shale rock (temperature is 383.15 K, pressure is 20 MPa, and the red line denotes the *Kn* = 0.1 threshold). (**a**) *Kn* of CH_4_ at varying pore diameter; (**b**) *Kn* of CO_2_ at varying pore diameter.

Based on Equations (4) and (5), the mean free paths of different gas molecules under varying temperature and pressure conditions can be calculated, as presented in Table 7. Meanwhile, the Knudsen numbers corresponding to gas diffusion in pores of different diameter can also be obtained, as shown in Figure 6. It can be seen from Table 7 that at a temperature of 383.15 K and a pressure of 20 MPa, CH_4_ diffusion is dominated by Fick diffusion in reservoir pores with a diameter greater than 3.91 nm (corresponding to *Kn* ≤ 0.1), while in reservoir pores with a diameter less than 3.91 nm, CH_4_ diffusion is dominated by Knudsen diffusion. Since the effective molecular diameter of CO_2_ differs from that of CH_4_, under the same temperature and pressure conditions (383.15 K, 20 MPa), CO_2_ diffusion is dominated by Fick diffusion in reservoir pores larger than 5.18 nm, while Knudsen diffusion becomes the dominant mechanism for CO_2_ in pores smaller than 5.18 nm.

Table 4 further reveals that under constant temperature conditions, the critical pore diameters separating Fick and Knudsen diffusion for both gases gradually decrease with increasing pressure. This is because higher pressure reduces the intermolecular distance and intensifies intermolecular collisions, which in turn shortens the mean free path of gas molecules, leading to a corresponding reduction in the critical pore diameter. Conversely, under constant pressure conditions, the critical pore diameters separating Fick and Knudsen diffusion for both gases gradually increase with rising temperature. This is attributed to the fact that higher temperature intensifies the thermal motion of gas molecules, thereby increasing their mean free path and resulting in a corresponding increase in the critical pore diameter.

**Figure 7:**
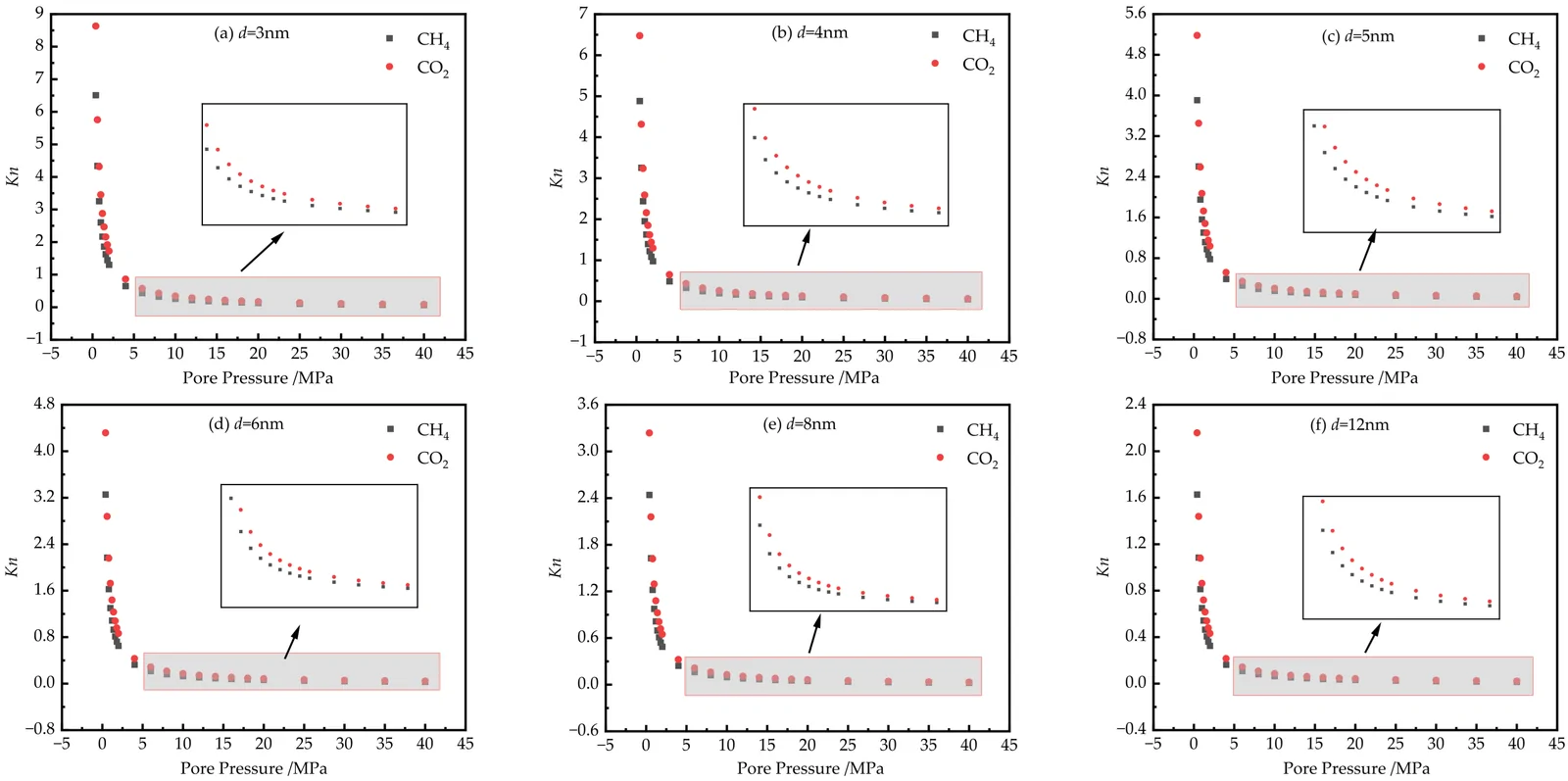
Variation in *Kn* with pressure for gas diffusion in pores of different sizes (temperature is 383.15 K). (**a**) Variation of *Kn* with pore pressure in 3 nm diameter pores; (**b**) Variation of *Kn* with pore pressure in 4 nm diameter pores; (**c**) Variation of *Kn* with pore pressure in 5 nm diameter pores; (**d**) Variation of *Kn* with pore pressure in 6 nm diameter pores; (**e**) Variation of *Kn* with pore pressure in 8 nm diameter pores; (**f**) Variation of *Kn* with pore pressure in 12 nm diameter pores.

Figure 7 presents the variations in Knudsen numbers for CH_4_ and CO_2_ with pressures in variable pore sizes. It is obvious that the collisions between gas molecules and pore walls in nanopores are significantly suppressed under high-pressure conditions [35,36,37], which is more favorable for gas diffusion in the reservoir. For a given pore size under identical temperature and pressure conditions, CO_2_ exhibits a larger Knudsen than CH_4_ due to its smaller molecular size.

When multiple bulk diffusion mechanisms coexist in rock pores, the corresponding diffusion coefficient can be calculated using the following comprehensive diffusion coefficient model [33,36,38]:

(6) $$D_{\mathrm{bulk}}=\frac{\lambda }{2r+\lambda }D_{\mathrm{knudsen}}+\frac{2r}{2r+\lambda }D_{\mathrm{fick}}$$

where

(7) $$D_{\mathrm{kundsen}}=\xi_{\mathrm{mb}}\frac{2r}{3}\sqrt{\frac{8RT}{\pi M}}$$

(8) $$D_{\mathrm{fick}}=\xi_{\mathrm{mb}}\frac{k_{b}T}{3\pi \mu_{g}\delta }$$

where *D*_knudsen_, *D*_fick_, *D*_bulk_ represent the Knudsen diffusion coefficient, Fick diffusion coefficient, and bulk diffusion coefficient, respectively, m^2^/s; *r* is the pore radius of the rock sample, m; *R* is the universal gas constant, 8.314 J/(mol·K); *M* is the gas molar mass, kg/mol; *µ*_g_ is the gas viscosity, Pa·s; *ξ*_mb_ is the dimensionless correction factor for bulk diffusion, which accounts for the effects of porosity, pore tortuosity, and the adsorption layer effect in shales. It can be expressed as follows:

(9) $$\xi_{\mathrm{mb}}=\frac{\phi }{\tau }{\left(1-\frac{d_{M}}{r}\right)}^{2}$$

where ϕ is the porosity, fraction; *τ* is the pore tortuosity, dimensionless; *d*_M_ is the gas molecular collision diameter, m.

It should be noted that the pore radius in the above equation is not a constant value. Under effective stress, the pores in shale samples undergo compressive deformation, which consequently causes variations in pore size. The connected pore volume in cylindrical shale samples can be conceptualized as a bundle of tortuous capillaries, and the effective cross-sectional area of this tortuous capillary bundle decreases with increasing effective stress [39,40,41,42]. The pore radius under the influence of effective stress can be expressed by Equation (10) [43]:

(10) $$r=\frac{r_{0}}{\sqrt{1+\frac{\alpha }{\phi K}(p_{\mathrm{confine}}-p)-\frac{\varepsilon_{L}p}{p+p_{L}}}}$$

where *r*_0_ is the initial pore radius, m; *r* is the pore radius under effective stress, m; *p*_confine_ and *p* are the confining pressure and pore pressure, respectively, Pa; *α* is the Biot coefficient, dimensionless; ϕ is the average porosity of the rock sample, dimensionless; *K* is the bulk modulus of the rock sample, Pa; $\varepsilon_{L}$ is the Langmuir volumetric strain constant; and *p*_L_ is the Langmuir pressure, Pa.

### 4.2. Surface Diffusion Coefficient

In shale gas reservoirs, gas is stored in both free and adsorbed states, with a dynamic equilibrium between the adsorbed phase and the bulk gas phase [44,45]. Based on the Langmuir adsorption model, the adsorbed gas concentration can be expressed by the following equation [46]:

(11) $$C_{s}=\frac{4\theta M}{\pi \delta^{3}N_{A}}$$

where *C*_s_ is the adsorbed gas concentration, kg/m^3^; *N*_A_ is Avogadro’s constant, 6.0221415 × 10^23^ mol^−1^; *θ* is the dimensionless gas coverage on pore wall surface, which can be determined by

(12) $$\theta =\frac{p}{p+p_{L}}$$

Driven by the chemical potential gradient, the diffusion coefficient of adsorbed gas on the pore wall surface can be calculated by the following equation [33,36].

(13) $$D_{\mathrm{surface}}=8.29\times 10^{-7}T^{0.5}\exp\left(-\frac{\Delta H^{0.8}}{RT}\right)\frac{(1-\theta )+\frac{\kappa }{2}\theta (2-\theta )+[H(1-\kappa )](1-\kappa )\frac{\kappa }{2}\theta^{2}}{{\left(1-\theta +\frac{\kappa }{2}\theta \right)}^{2}}\xi_{\mathrm{ms}}\xi_{\mathrm{real}}$$

where *D*_surface_ is the surface diffusion coefficient, m^2^/s; Δ*H* is the isosteric heat of adsorption, J/mol; *κ* is the molecular blocking coefficient, which is defined as the ratio of the blocking rate coefficient to the forward rate coefficient, dimensionless; *H*(1 − *κ*) is the Heaviside function determined by

(14) $$H(1-\kappa )=\left\{\begin{array}{ll} 0, & \kappa >1\\ 1, & 0\leq \kappa \leq 1 \end{array}\right.$$

*ξ*_ms_ in Equation (13) is the dimensionless correction factor for surface diffusion, which accounts for the effects of pore turtuosity and the effective diffusion area of molecules within the adsorption layer. It can be determined by the following equation [47]:

(15) $$\xi_{\mathrm{ms}}=\xi_{\mathrm{mb}}\left[{\left(1-\frac{d_{M}}{r}\right)}^{-2}-1\right]$$

*ξ*_real_ in Equation (13) is the dimensionless correction factor for real surface diffusion, which accounts for the effect of the discontinuous distribution of organic matter on pore wall surfaces. As recommended by Zhong et al. [36], its value ranges from 10^−3^ to 10^−1^, and the specific value can be determined by fitting with experimental data.

### 4.3. Total Gas Diffusion Coefficient

Considering the combined effects of bulk gas diffusion and surface diffusion, the total gas diffusion coefficient in the pores of shale reservoirs can be expressed as follows:

(16) $$D_{\mathrm{total}}=D_{\mathrm{bulk}}+D_{\mathrm{surface}}$$

Substitution of Equations (6)–(8) and (13) into Equation (16) yields

(17) $$\begin{array}{c} D_{\mathrm{total}}=\left[8.29\times 10^{-7}T^{0.5}\exp\left(-\frac{\Delta H^{0.8}}{RT}\right)\frac{(1-\theta )\frac{\kappa }{2}\theta (2-\theta )+[H(1-\kappa )](1-\kappa )\frac{\kappa }{2}\theta^{2}}{{\left(1-\theta +\frac{\kappa }{2}\theta \right)}^{2}}\right]\times \xi_{\mathrm{ms}}\xi_{\mathrm{real}}\\ +\left[\frac{\frac{\kappa_{b}T}{\sqrt{2}\pi \delta^{2}p}}{2+\frac{\kappa_{b}T}{\sqrt{2}\pi \delta^{2}p}}\times \frac{2r}{3}\sqrt{\frac{8RT}{\pi M}}+\frac{2r}{2+\frac{\kappa_{b}T}{\sqrt{2\pi \delta^{2}p}}}\times \frac{\kappa_{b}T}{3\pi \mu_{g}\delta }\right]\xi_{\mathrm{mb}} \end{array}$$

### 4.4. Model Calibration

**Figure 8:**
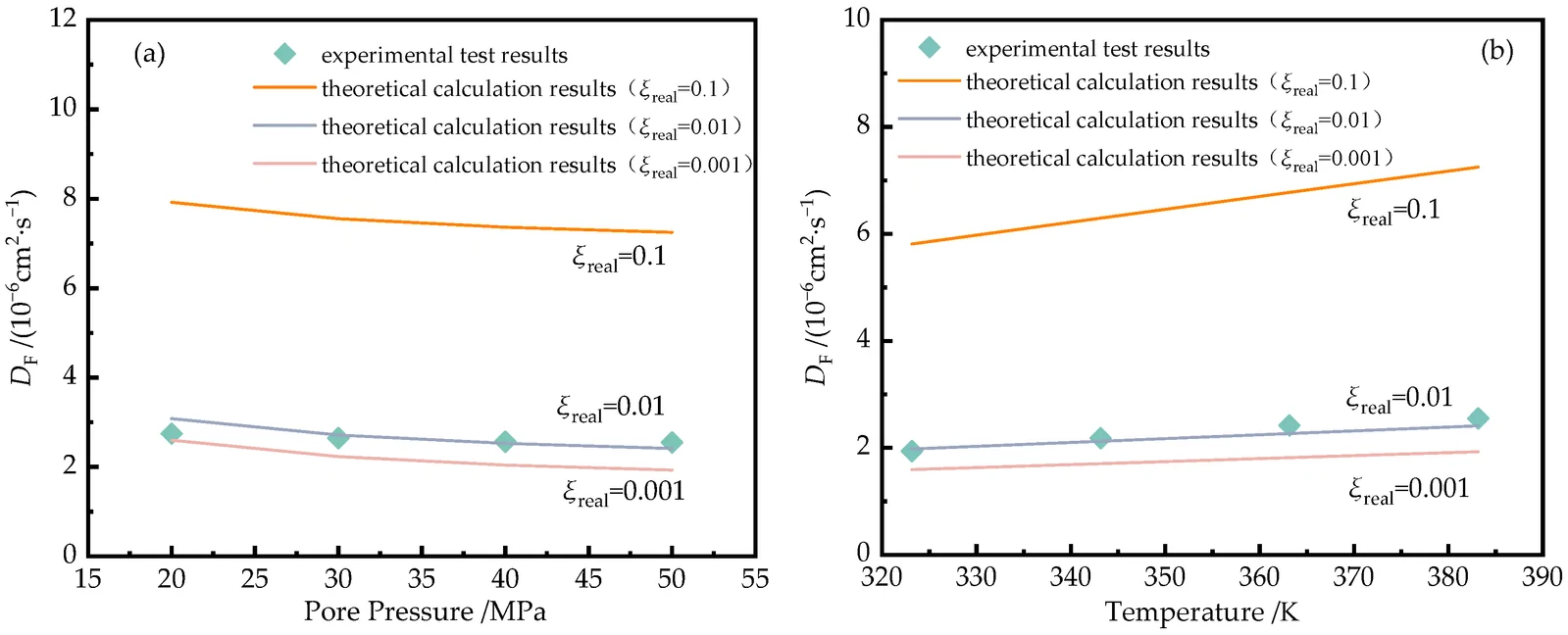
Comparison between the calculated and experimentally measured CH_4_ diffusion coefficients. (**a**) Under different pressures (temperature = 383.15 K); (**b**) under different temperatures (pore pressure = 50 MPa).

**Figure 9:**
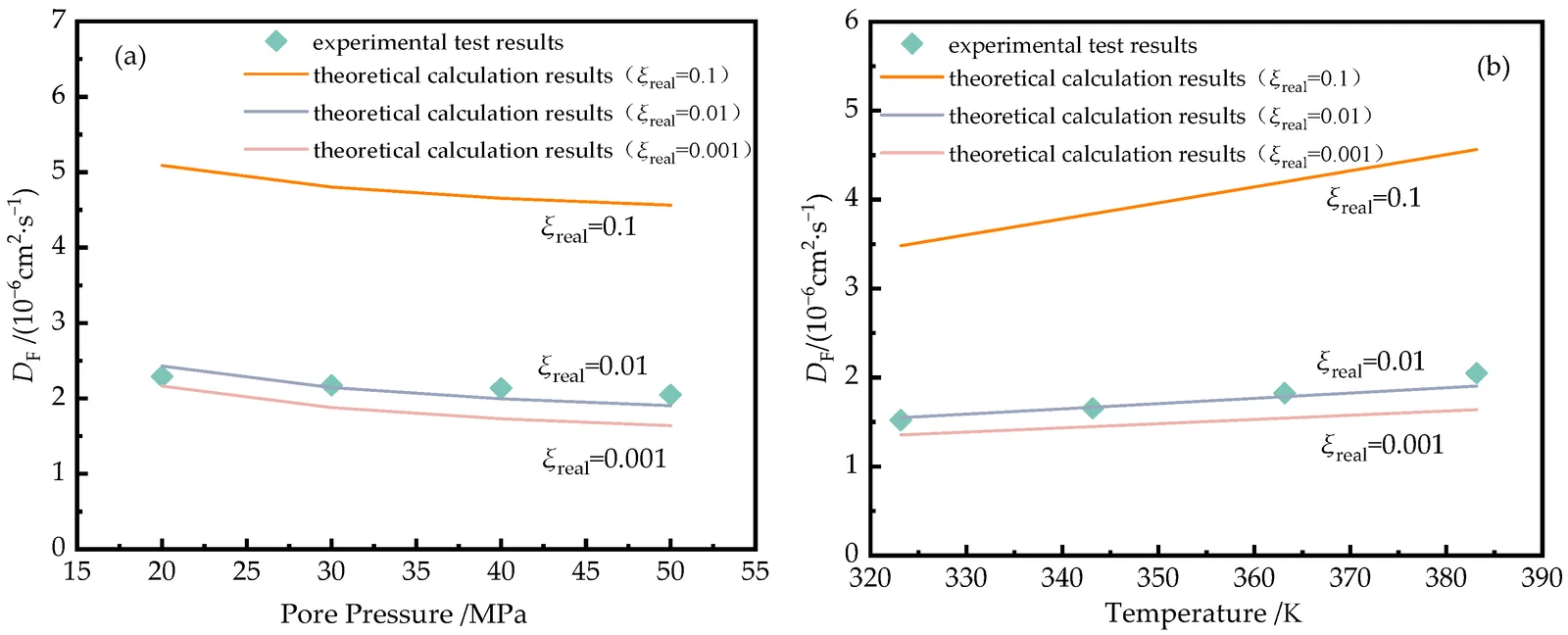
Comparison between the calculated and experimentally measured CO_2_ diffusion coefficients. (**a**) Under different pressures (temperature = 383.15 K); (**b**) under different temperatures (pore pressure = 50 MPa).

Using the total diffusion coefficient formula (Equation 17), the diffusion coefficients of CH_4_ and CO_2_ in the typical shale sample under the aforementioned experimental pressure and temperature conditions were calculated. The parameters used in the calculation are listed in Table 8, and the corresponding results are presented in Figure 8 and Figure 9.

Figure 8 and Figure 9 compare the calculated gas diffusion coefficients under different pressures and temperatures with experimentally measured results for both gases. It can be observed that, for different values of *ξ*_real_, the variation trends of the calculated *D*_total_ with increasing pressure and temperature are fully consistent with the experimental data. The total diffusion coefficient decreases with rising pressure, while it increases as the temperature increases.

Further comparison between the calculated diffusion coefficients and the experimentally measured results under different values of *ξ*_real_ (Table 9) shows that when *ξ*_real_ is set to 0.01, the errors between the calculated and experimentally measured values for CH_4_ and CO_2_ are the smallest, which are 2.03% and −2.24%, respectively. It should be mentioned that the parameter *ξ*_real_ is obtained by fitting the theoretical model to the measured experimental data, and the two error values are the fitting residuals under the current dataset, not independent validation results. Further independent experimental data are required for strict model validation in future work.

It should also be addressed that *ξ*_real_ is not a purely empirical fitting parameter; it is actually a parameter characterizing the effective connectivity of organic pores in shale matrix. Since organic matter only accounts for a small proportion of the shale matrix volume, the surface diffusion paths of adsorbed gas in shale organic pores are spatially discontinuous, rather than the ideal continuous distribution assumed in classical surface diffusion theory. The parameter *ξ*_real_ corrects the overestimated theoretical surface diffusion coefficient derived from the continuous organic medium assumption, making the model consistent with the actual diffusion behavior in heterogeneous shales. Considering that direct experimental measurement of organic pore effective connectivity in shale matrix is extremely difficult, we determined the value of *ξ*_real_ by fitting the theoretical model against the experimental data. According to our fitting results, *ξ*_real_ = 0.01 achieves the optimal fitting performance under all investigated temperature, pressure, and pore-size conditions in this work. Under the constant effective stress experimental design, the spatial distribution and volume fraction of organic matter in the shale matrix remain stable, and the surface diffusion path discontinuity will not vary with temperature, pressure or pore size variations within our test range.

It is worth noting that this correction parameter is strongly dependent on pore and mineral properties of shale. Zhong et al. [36] adopted the unified apparent-diffusion-coefficient model to fit experimental results of black shale samples collected from the Longmaxi Formation, and obtained an optimal real surface diffusion correction factor of 0.01, which shows high consistency with the *ξ*_real_ value fitted in this study. Zou et al. [6]. carried out methane diffusion experiments on shale core samples taken from the Niutitang Formation, and determined that the correction coefficient ranges from 0.001 to 0.01. This result further verifies the inherent sample- and condition-dependence of parameter *ξ*_real_.

The comparison results demonstrate that the total diffusion coefficient formula can accurately characterize the gas diffusion behaviors in shale samples collected from the Longmaxi Formation under high-temperature and high-pressure conditions, enabling further sensitivity analysis on this basis.

### 4.5. Influencing Factors of Gas Diffusion Behavior Under High-Temperature High-Pressure Conditions

**Figure 10:**
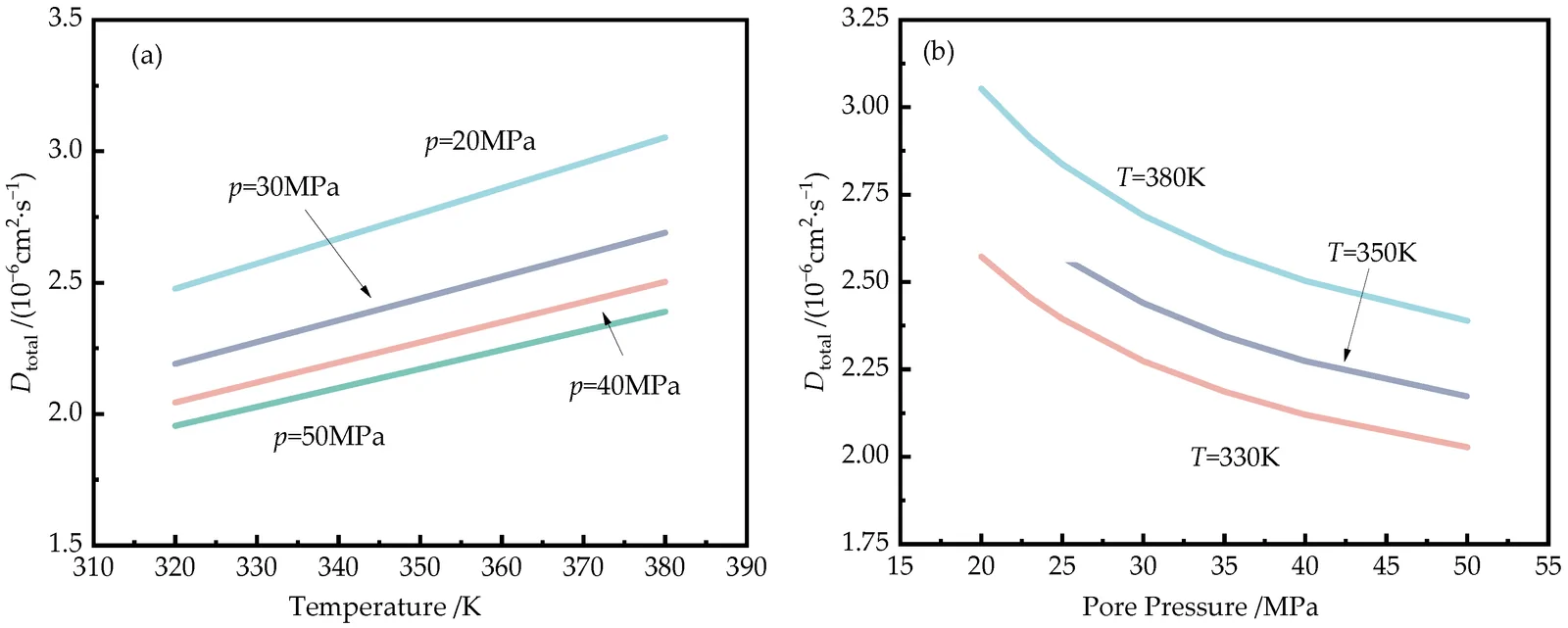

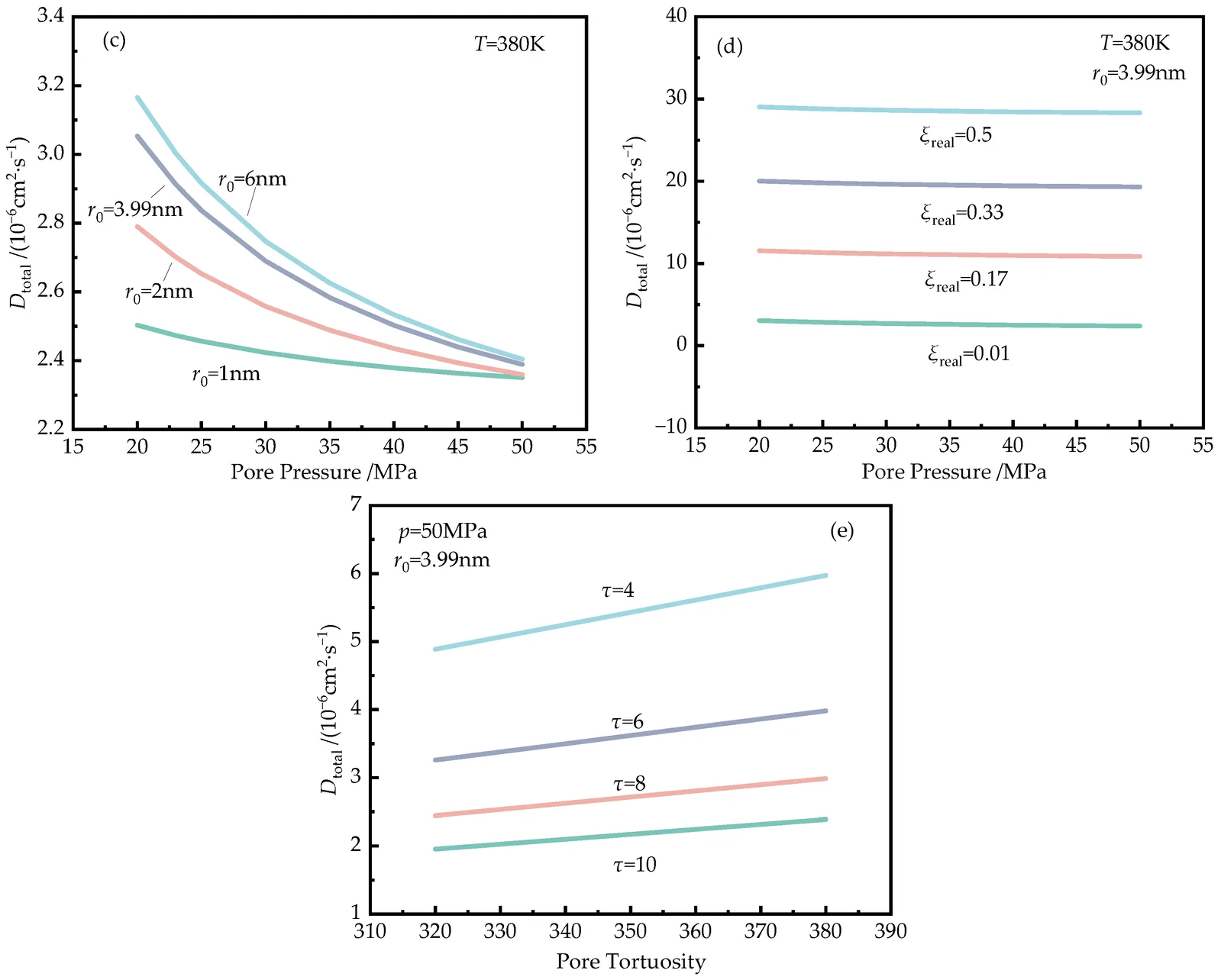
Variation curves of CH_4_ diffusion coefficient. (**a**) Variation in total diffusion coefficient with temperature. (**b**) Variation in total diffusion coefficient with pressure. (**c**) Variation in total diffusion coefficient with pore size. (**d**) Variation in total diffusion coefficient with correction coefficient. (**e**) Variation in total diffusion coefficient with pore tortuosity.

**Figure 11:**
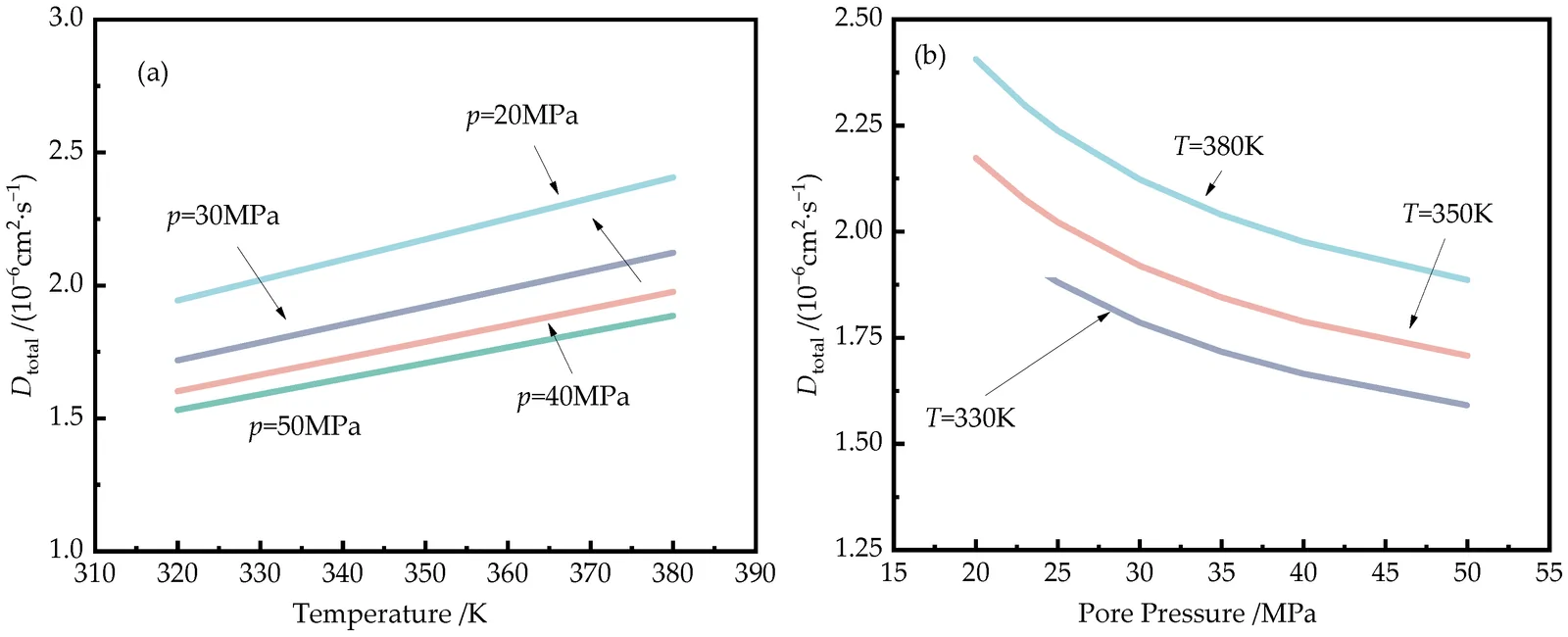

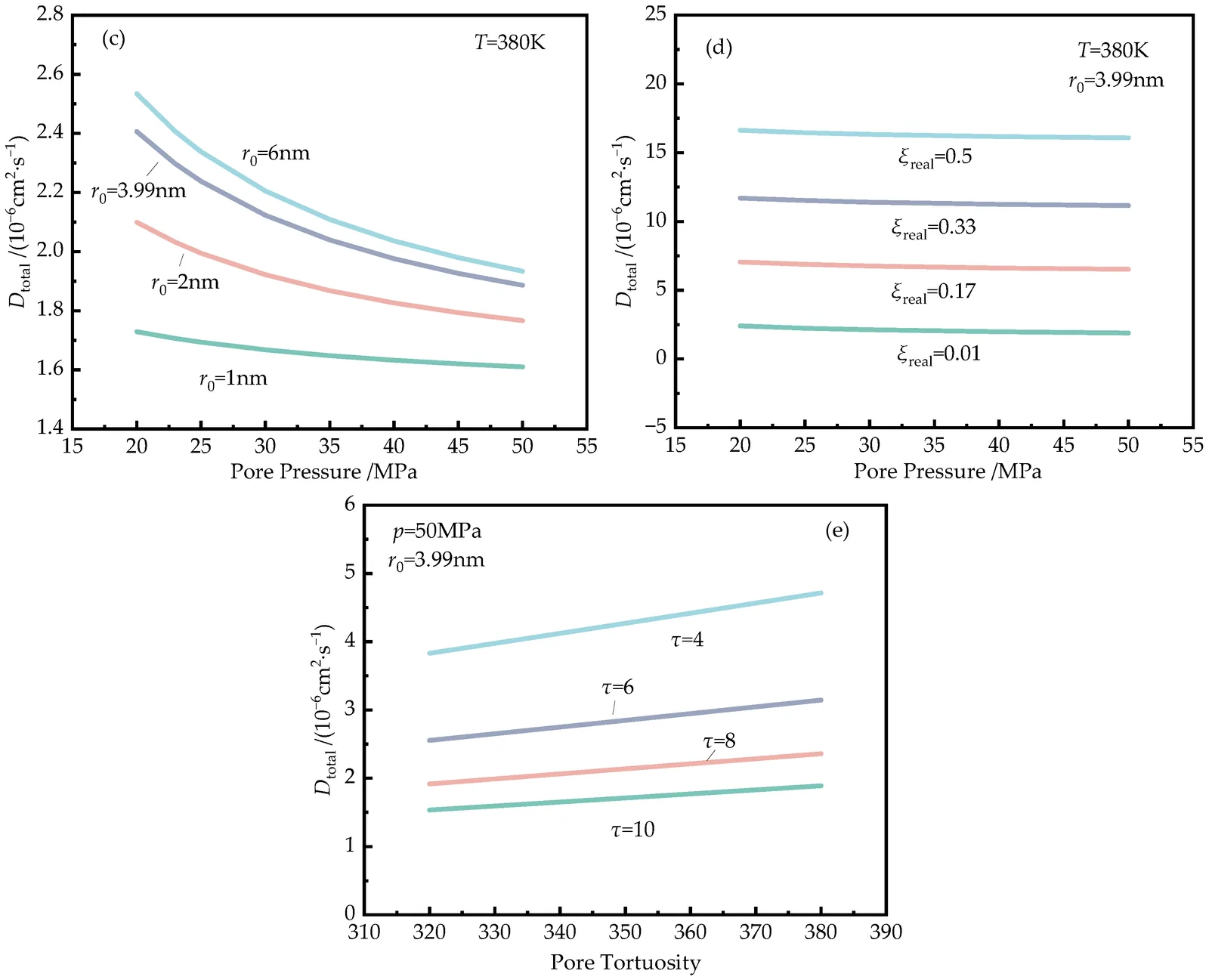
Variation curves of CO_2_ diffusion coefficient. (**a**) Variation in total diffusion coefficient with temperature. (**b**) Variation in total diffusion coefficient with pressure. (**c**) Variation in total diffusion coefficient with pore size. (**d**) Variation in total diffusion coefficient with correction coefficient. (**e**) Variation in total diffusion coefficient with pore tortuosity.

**Figure 12:**
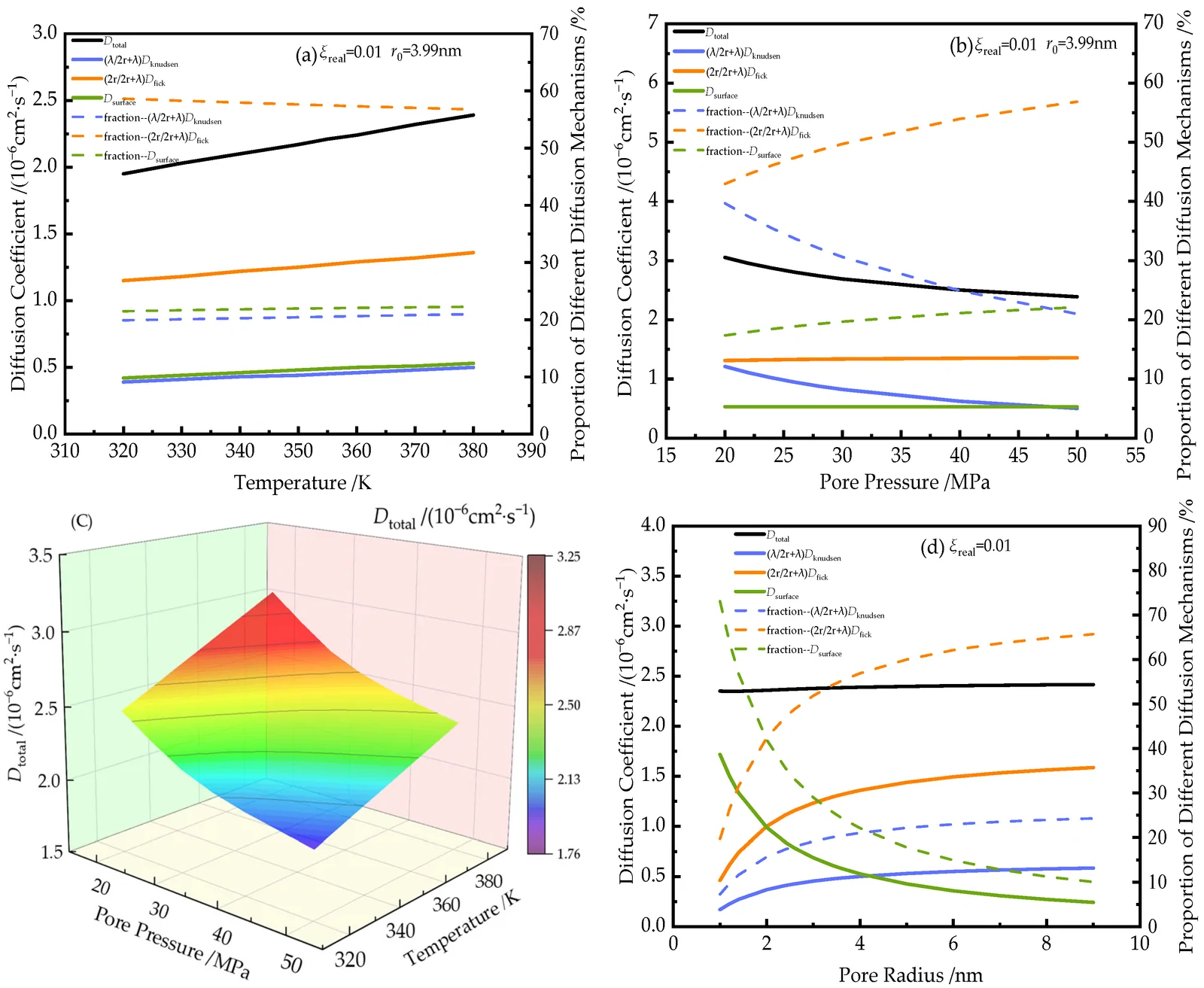
Variation in the contribution proportions of different diffusion mechanisms for CH_4_. (**a**) Under varying temperatures (pressure = 50 MPa). (**b**) Under varying pressures (temperature = 380 K). (**c**) Coupled temperature–pressure dependence of the total diffusion coefficient. (**d**) Within varying pore radius (temperature = 380 K, pressure = 50 MPa).

**Figure 13:**
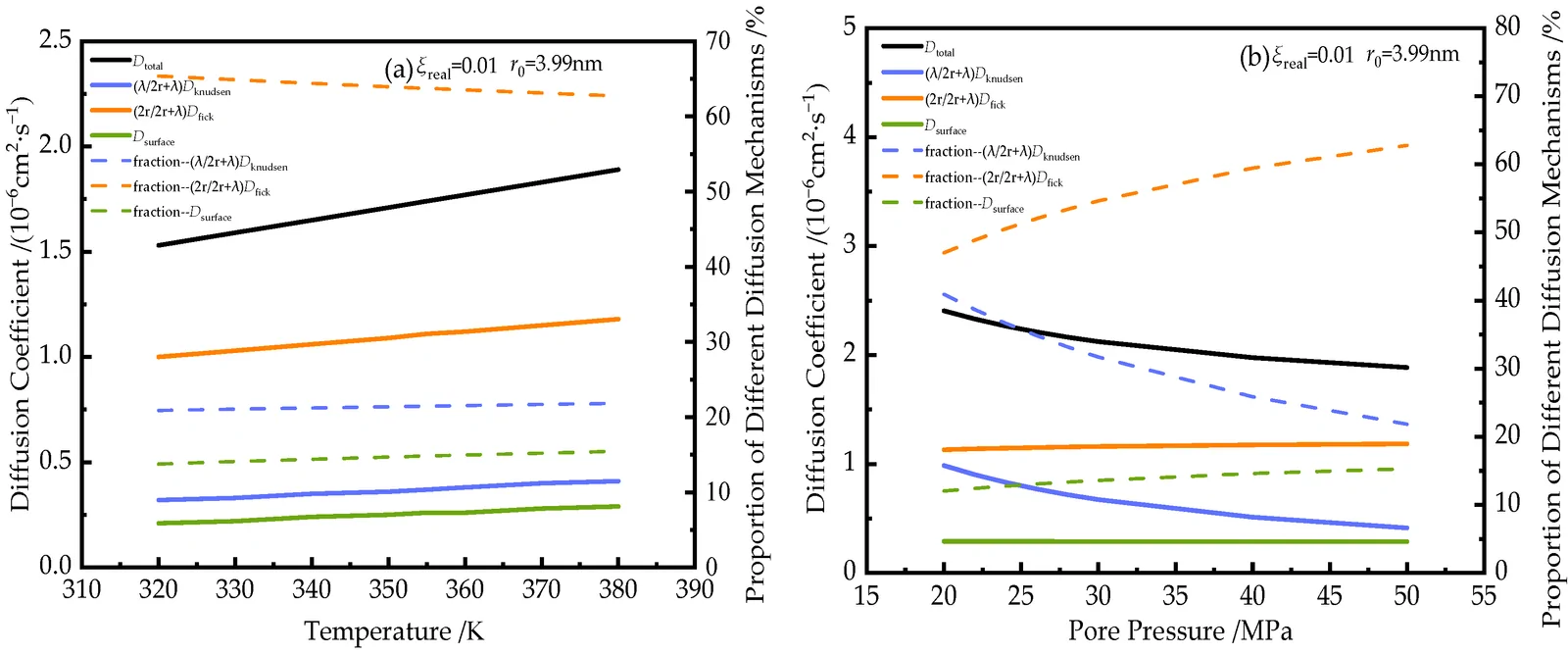

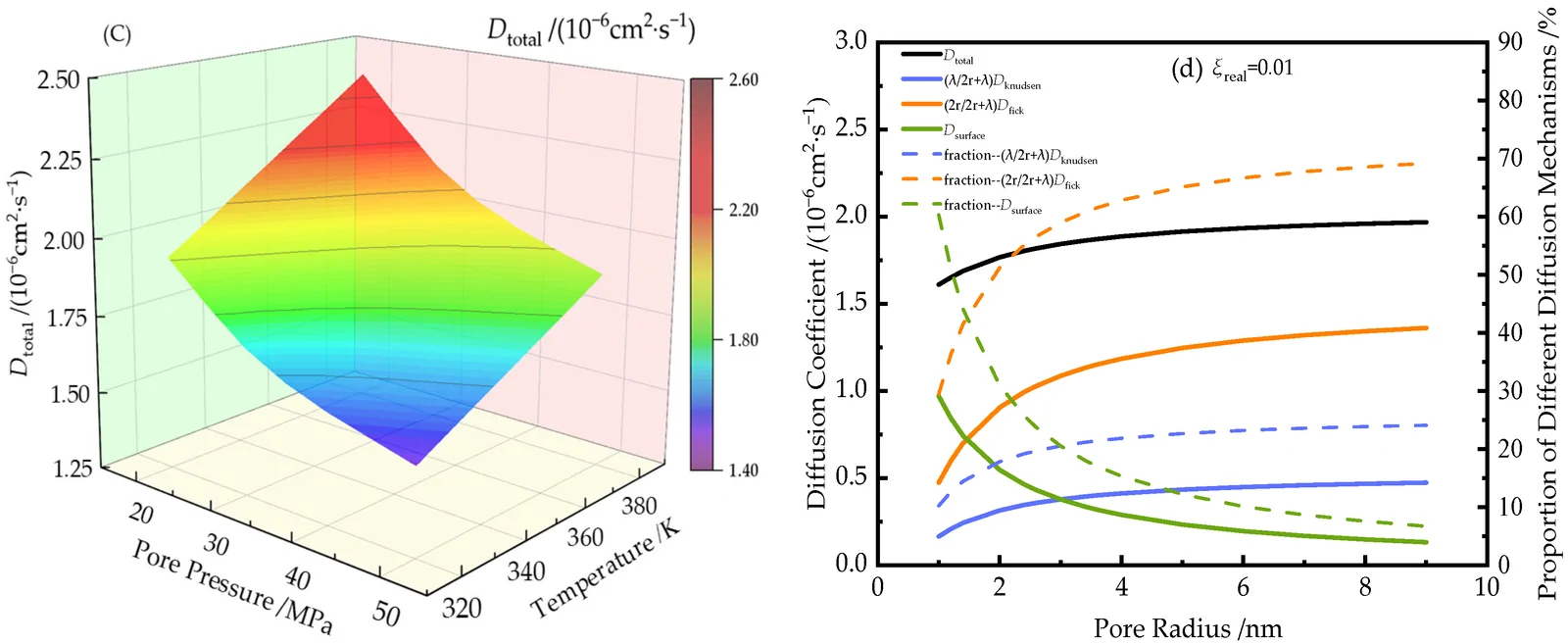
Variation in the contribution proportions of different diffusion mechanisms for CO_2_. (**a**) Under varying temperatures (pressure = 50 MPa). (**b**) Under varying pressures (temperature = 380 K). (**c**) Coupled temperature–pressure dependence of the total diffusion coefficient. (**d**) Within varying pore radius (temperature = 380 K, pressure = 50 MPa).

Gas diffusion in shale reservoirs is governed by the combined effects of multiple factors, including temperature, pressure and pore radius. To quantitatively clarify the influence of relevant parameters on the diffusion behaviors of CH_4_ and CO_2_ in shale reservoirs, a series of diffusion coefficient calculations were performed via the controlled variable method. Based on the matching results presented in Section 4.4, the value of the correction factor *ξ*_real_ adopted in the following sensitivity analysis is specified as 0.01. The variation range of key relevant parameters and the total diffusion coefficients obtained from the calculation are presented in Table 10 and Table 11 and Figure 10 and Figure 11. Furthermore, the contribution proportions of different diffusion mechanisms under varying pressure and temperature conditions are presented in Figure 12 and Figure 13.

It should be clarified that the individual contribution proportions of different diffusion mechanisms presented in Figure 12 and Figure 13 are not directly measured via experiments, but quantitatively decomposed using the calibrated theoretical model.

As illustrated in Figure 10a and Figure 11a, Figure 12a, and Figure 13a, increasing temperature enhances both bulk diffusion and surface diffusion processes. This phenomenon can be attributed to the fact that higher temperature enhances the kinetic energy of gas molecules, thereby raising the diffusion coefficient corresponding to each individual diffusion mechanism. Furthermore, the quantitative results presented in Table 10 and Table 11 demonstrate that, for both CH_4_ and CO_2_, the temperature sensitivity of Fick diffusion and surface diffusion is significantly stronger than that of Knudsen diffusion. When temperature increases from 330 K to 380 K, the Fick diffusion coefficient of CH_4_ (i.e., $D_{\mathrm{fick}}$) increases from 1.21 × 10^−6^ cm^2^**·**s^−1^ to 1.39 × 10^−6^ cm^2^**·**s^−1^, corresponding to a percentage increment of 14.88%. The percentage increase in the surface diffusion *D*_surface_ is 20.45%, while Knudsen diffusion coefficient ($D_{\mathrm{knudsen}}$) only exhibits an 8.14% increment under the same temperature change. Table 11 presents the corresponding results for CO_2_, which exhibits a similar variation trend.

Additionally, it can also be observed from Figure 12a and Figure 13a that for the fixed pore radius (*r* = 3.99 nm, the average pore radius of the tested shale sample), Fick diffusion constitutes a substantial proportion of the overall gas diffusion across the simulated conditions, despite a slight reduction in its relative contribution as temperature increases.

Figure 10b and Figure 11b, Figure 12b and Figure 13b demonstrate that the diffusion coefficients of CH_4_ and CO_2_ exhibit obvious pressure dependence as the pressure varies from 20 MPa to 50 MPa. It is observed that the total diffusion coefficient decreases gradually with increasing pressure. When the pressure is below 30 MPa, the diffusion coefficient shows a more pronounced variation with pressure (Figure 10b and Figure 11b); once the pressure exceeds 30 MPa, the magnitude of the reduction in the total diffusion coefficient gradually diminishes with further pressure rise. Meanwhile, the relative contributions of different diffusion mechanisms to the total diffusion coefficient vary systematically with pressure. As demonstrated in Figure 12b and Figure 13b, the effective pore size of shale increases with rising pore pressure. The weighted Knudsen diffusion coefficient (i.e., $\frac{\lambda }{2r+\lambda }D_{\mathrm{knudsen}}$) decreases significantly as pressure increases, which is the primary reason of the overall decline in the total diffusion coefficient. The proportion of Knudsen diffusion in total diffusion also gradually diminishes with increasing pressure. By contrast, the contribution proportion of Fick diffusion increases notably, while pressure exerts a relatively minor effect on the surface diffusion coefficient.

Figure 12c and Figure 13c illustrate the variation in total diffusion coefficient of CH_4_ and CO_2_ under the coupled effect of temperature and pressure. It is clear that under high-temperature and high-pressure conditions, the gas diffusion coefficient for both CH_4_ and CO_2_ in shale pores is more sensitive to pressure than to temperature.

Figure 10c and Figure 11c illustrate the influence of pore size on the diffusion coefficients of CH_4_ and CO_2_ in shales. Larger pore diameters provide more free volume for bulk diffusion, which in turn leads to an obvious increase in the total diffusion coefficient. Furthermore, Figure 12d and Figure 13d indicate that with the increase in pore radius, the relative contribution of surface diffusion to the total diffusion coefficient gradually decreases, which can be explained by the decline in the volume fraction of the adsorption layer to the total pore cross-section.

Table 12 compares the measured diffusion coefficients of CH_4_ and CO_2_ in shales obtained in this work with those reported in the literature for similar problems. The diffusion coefficients in this study fall within the same order of magnitude as previously published molecular simulation and experimental results. The minor deviations across datasets primarily originate from inherent differences in shale pore structure, in situ temperature–pressure conditions, and total organic matter (TOC) composition of different samples, which further validates the reliability of our study.

## 5. Conclusions

In this work, high-temperature and high-pressure experimental investigations were performed to investigate the diffusion behaviors of supercritical CH_4_ and CO_2_ in core samples collected from the Longmaxi Formation shale in the Sichuan Basin, China.

The experimental results show that under the pressure range of 20–50 MPa and temperature range of 323.15–383.15 K, Fick diffusion constitutes a substantial proportion of the total gas diffusion. Under identical temperature and pressure conditions, the diffusion coefficient of CH_4_ in shale nanopores is higher than that of CO_2_. The diffusion coefficients of both CH_4_ and CO_2_ in shales show an approximately linear increasing trend with rising temperature, while they decrease progressively as pressure increases, with the decline rate gradually diminishing at higher pressure levels.

A weighted total diffusion coefficient model that comprehensively incorporates both bulk diffusion and surface diffusion mechanisms was adopted to regress the experimental data and to screen the controlling factors of gas diffusion in shales. The value of the in situ correction factor *ξ*_real_ for Longmaxi Formation shale was determined based on the matching degree between the theoretically calculated and experimentally measured diffusion coefficient profiles.

Quantitative theoretical analysis indicates the relative contribution of each individual diffusion mechanism varies under different temperature and pressure conditions. Fick diffusion constitutes a substantial proportion of the total gas diffusion for both CH_4_ and CO_2_ within the shale sample under high temperature and high pressure conditions. An increase in temperature enhances both bulk diffusion and surface diffusion, while the relative contribution of Fick diffusion gradually decreases with rising temperature. Increasing pressure would lead to the decrease in total diffusion coefficient but increase the contribution percentage of Fick diffusion. Under coupled effects of high pressure and high temperature, the total diffusion coefficient is more sensitive to pressure than to temperature. Increasing pore size significantly strengthens bulk diffusion and slightly restricts the contribution of surface diffusion, yet leads to an increase in the total diffusion coefficient.

The findings of this study can offer direct insights for optimizing shale gas development and CO_2_ enhanced shale gas recovery (CO_2_-EGR), and evaluating the feasibility of underground gas storage (UGS) in depleted shale gas reservoirs. The experimentally measured diffusion coefficients can be directly incorporated into reservoir numerical simulators to correct the traditional seepage-dominated transport models, thereby significantly improving the accuracy of long-term production dynamics prediction and ultimate recovery evaluation. Furthermore, in the context of CO_2_-EGR or UGS, the quantified diffusion coefficients for both CO_2_ and CH_4_ enable a more precise evaluation of the long-term migration and distribution of injected CO_2_ in shale formations, which is crucial for reducing the risk of CO_2_ leakage and enhancing the storage safety and sequestration efficiency.

Although this work systematically investigates the diffusion behaviors of CO_2_ and CH_4_ in shale nanopores under varying pressure and temperature conditions, several limitations should be noted. First, the diffusion coefficients were measured based on one representative shale core sample, which does not fully account for the strong heterogeneity inherent to shale formations. Second, the study focuses on single-component gas (pure CH_4_ and pure CO_2_) diffusion, without addressing the competitive adsorption and diffusion phenomena that occur in multi-component gas mixtures-a critical factor influencing gas transport efficiency in actual CO_2_-EGR processes. Third, constrained by the experimental apparatus, the investigated pressure range does not fully cover the entire spectrum of in situ reservoir conditions, particularly the ultra-high pressures encountered in deep shale formations.

To address these limitations and build upon the current findings, future studies can focus on (1) conducting multi-component gas mixture diffusion experiments to reveal competitive gas transport mechanisms and provide essential parameters for predicting gas recovery and CO_2_ storage performance in actual shale plays; (2) expanding the study to include a diverse set of shale core samples with varying pore structures and mineral compositions to better represent reservoir heterogeneity; (3) establishing a pore-size-distribution weighted diffusion model to enhance the accuracy of theoretical predictions across different shale types; and (4) conducting diffusion coefficient measurements under ultra-high pressure conditions to more accurately evaluate in situ gas diffusion behaviors in deep shale formations.

## Figures and Tables

**Table 1:** Basic parameters of core samples.

Length (cm)	Diameter (cm)	Permeability (mD)	Porosity (%)
5.074	2.522	0.0187	1.8

**Table 2:** Mineral composition and TOC of the shale core sample.

Mineral Component (wt%)						TOC (wt%)
Quartz	Plagioclase	Calcite	Dolomite	Pyrite	Clays	
67.7	3.3	14.2	9.6	0.8	4.4	3.45

**Table 3:** Clay mineral compositions of the shale core sample.

**Clay Mineral Component (wt%)**		
**Illite**	**Illite–Smectite Mixed Layer (I/S)**	**Chlorite**
89.0	7.6	3.4

**Table 4:** Standard crystallographic parameters of all identified main mineral phases.

Mineral Name	Crystal System	Space Group	Lattice Parameter (Å)			Interaxial Angle (°)			Number of Formula Units per Cell Z
			*a*	*b*	*c*	*α*	*β*	*γ*	
Quartz	Trigonal	P3_1_21	4.913	4.913	5.405	90	90	120	3
Plagioclase (Albite end-member)	Triclinic	C1̅	8.137	12.787	7.158	93.47	116.25	90.27	4
Calcite	Trigonal	R3̅c	4.989	4.989	17.068	90	90	120	6
Dolomite	Trigonal	R3̅	4.808	4.808	16.022	90	90	120	3
Pyrite	Isometric	Pa3̅	5.417	5.417	5.417	90	90	90	4
Illite	Monoclinic	C2/m	5.200	9.000	10.000	90	101.5	90	2
Chlorite	Monoclinic	C2/m	5.320	9.210	14.370	90	97.0	90	2
Illite–smectite mixes layer	Monoclinic	C2/m	Variable with I/S ratio			90	~99	90	2

**Table 5:** Measured diffusion coefficients of CH_4_ and CO_2_ at different temperatures.

Gas	Pore Pressure (MPa)	Confining Pressure (MPa)	Temperature (K)	Diffusion Coefficient (10^−6^ cm^2^·s^−1^)
CH_4_	50	60	323.15	1.938
			343.15	2.179
			363.15	2.420
			383.15	2.549
CO_2_	50	60	323.15	1.521
			343.15	1.654
			363.15	1.824
			383.15	2.049

**Table 6:** Measured diffusion coefficients of CH_4_ and CO_2_ at different pressures.

Gas	Temperature (K)	Pore Pressure (MPa)	Confining Pressure (MPa)	Diffusion Coefficient (10^−6^ cm^2^·s^−1^)
CH_4_	383.15	20	30	2.744
		30	40	2.647
		40	50	2.563
		50	60	2.549
CO_2_	383.15	20	30	2.291
		30	40	2.173
		40	50	2.135
		50	60	2.049

**Table 7:** Calculation results of the mean free path of gas molecules under varying temperature and pressure conditions.

Temperature (K)	Pore Pressure (MPa)	Effective Molecular Diameter (nm)		Mean Free Path of Gas Molecule (nm)		Critical Pore Diameter at *Kn* = 0.1 (nm)	
		CH_4_	CO_2_	CH_4_	CO_2_	CH_4_	CO_2_
323.15	20	0.380	0.330	0.329	0.437	3.29	4.37
	30			0.220	0.291	2.2	2.91
	40			0.165	0.218	1.65	2.18
	50			0.132	0.175	1.32	1.75
343.15	20			0.350	0.464	3.5	4.64
	30			0.233	0.309	2.33	3.09
	40			0.175	0.232	1.75	2.32
	50			0.140	0.186	1.4	1.86
363.15	20			0.370	0.491	3.7	4.91
	30			0.247	0.327	2.47	3.27
	40			0.185	0.245	1.85	2.45
	50			0.148	0.196	1.48	1.96
383.15	20			0.391	0.518	3.91	5.18
	30			0.260	0.345	2.6	3.45
	40			0.195	0.259	1.95	2.59
	50			0.156	0.207	1.56	2.07

**Table 8:** Parameters used in the calculation of diffusion coefficients for CH_4_ and CO_2_.

Parameters	Values	Parameters	Values
*T*/*K*	323.15~383.15	*K*/Pa	8 × 10^8^
*p*/Pa	(20~50) × 10^6^	*ξ*_real_	10^−3^~10^−1^
*p*_confine_*/*Pa	(30~60) × 10^6^	*k*_b_/*(*J·K⁻¹*)*	1.380 × 10^−23^
*R*/*(*J·mol^−1^·K^−1^*)*	8.314	*p*_L_*/*Pa	8 × 10^6^
*κ*	0.5	*r*_0_*/*m	3.99 × 10^−9^
*H*(1 *− κ*)	1	*α*	0.8
*τ*	10	*ε*_L_	0.05
ϕ	0.018	*δ*/*d*_M_/m	CH_4_: 3.8 × 10^−10^; CO_2_: 3.3 × 10^−10^
Δ*H*/*(*J·mol^−1^*)*	CH_4_: 18,000; CO_2_: 32,000	*M*/*(*kg·mol^−1^*)*	CH_4_: 0.016; CO_2_: 0.044
*μ*_g_/*(*Pa·s*)*	CH_4_: 0.000014; CO_2_: 0.000019		

**Table 9:** Error analysis between calculated and experimentally measured diffusion coefficients.

Pressure (MPa)	Error (%)					
	CH_4_			CO_2_		
	*ξ*_real_ = 0.1	*ξ*_real_ = 0.01	*ξ*_real_ = 0.001	*ξ*_real_ = 0.1	*ξ*_real_ = 0.01	*ξ*_real_ = 0.001
20	188.64	12.33	−5.31	122.14	6.04	−5.56
30	185.36	2.59	−15.69	121.04	−1.36	−13.6
40	187.35	−1.41	−20.29	118.02	−6.56	−19.01
50	184.42	−5.38	−24.36	122.73	−7.07	−20.05
Average	186.44	2.03	−16.41	120.98	−2.24	−14.56

**Table 10:** Variation ranges of relevant parameters and corresponding calculation results for CH_4_.

Variable		*D*_knudsen_ (10^−6^ cm^2^·s^−1^)	*D*_fick_ (10^−6^ cm^2^·s^−1^)	$\frac{\lambda }{2r+\lambda }$ *D*_knudsen_ (10^−6^ cm^2^·s^−1^)	$\frac{2r}{2r+\lambda }$ *D*_fick_ (10^−6^ cm^2^·s^−1^)	*D*_surface_ (10^−6^ cm^2^·s^−1^)	*D*_total_ (10^−6^ cm^2^·s^−1^)
Pressure (*T* = 370 K; *r*_0_ = 3.99 nm)	*p* = 20 MPa 50 MPa	21.07 21.07	1.35 1.35	1.16 0.48	1.28 1.32	0.51 0.51	2.96 2.32
Temperature (*p* = 50 MPa; *r*_0_ = 3.99 nm)	*T* = 330 K 380 K	19.90 21.52	1.21 1.39	0.41 0.50	1.18 1.36	0.44 0.53	2.03 2.39
Pore Size (*T* = 380 K; *p* = 50 MPa)	*r*_0_ = 1 nm 6 nm	1.93 35.05	0.50 1.52	0.17 0.55	0.46 1.49	1.72 0.36	2.35 2.40
Correction coefficient (*T* = 380 K; *p* = 50 MPa; *r*_0_ = 3.99 nm)	*ξ*_real_ = 0.5 0.01	21.36 21.52	1.39 1.39	0.50 0.50	1.36 1.36	26.59 0.53	28.45 2.39
Pore tortuosity (*T* = 380 K; *p* = 50 MPa; *r*_0_ = 3.99 nm)	*τ* = 4 10	53.80 21.52	3.48 1.39	1.25 0.50	3.40 1.36	1.32 0.53	5.97 2.39
Langmuir pressure constant (*T* = 380 K; *p* = 50 MPa; *r*_0_ = 3.99 nm)	*p*_L_ =5 × 10^6^ Pa 8 × 10^6^ Pa	21.54 21.52	1.39 1.39	0.50 0.50	1.36 1.36	0.53 0.53	2.38918 2.38915

**Table 11:** Variation ranges of relevant parameters and corresponding calculation results for CO_2_.

Variable		*D*_knudsen_ (10^−6^ cm^2^·s^−1^)	*D*_fick_ (10^−6^ cm^2^·s^−1^)	$\frac{\lambda }{2r+\lambda }$ *D*_knudsen_ (10^−6^ cm^2^·s^−1^)	$\frac{2r}{2r+\lambda }$ *D*_fick_ (10^−6^ cm^2^·s^−1^)	*D*_surface_ (10^−6^ cm^2^·s^−1^)	*D*_total_ (10^−6^ cm^2^·s^−1^)
Pressure (*T* = 370 K; *r*_0_ = 3.99 nm)	*p* = 20 MPa 50 MPa	13.22 13.26	1.19 1.19	0.95 0.4	1.10 1.15	0.28 0.28	2.33 1.83
Temperature (*p* = 50 MPa; *r*_0_ = 3.99 nm)	*T* = 330 K 380 K	12.52 13.43	1.06 1.22	0.34 0.41	1.03 1.18	0.22 0.29	1.59 1.89
Pore Size (*T* = 380 K; *p* = 50 MPa)	*r*_0_ = 1 nm 6 nm	1.47 21.76	0.53 1.32	0.17 0.45	0.47 1.29	0.97 0.20	1.61 1.93
Correction coefficient (*T* = 380 K; *p* = 50 MPa; *r*_0_ = 3.99 nm)	*ξ*_real_ = 0.5 0.01	13.43 13.43	1.22 1.22	0.41 0.41	1.18 1.18	14.48 0.29	16.08 1.89
Pore tortuosity (*T* = 380 K; *p* = 50 MPa; *r*_0_ = 3.99 nm)	*τ* = 4 10	33.58 13.43	3.05 1.22	1.03 0.41	2.96 1.18	0.72 0.29	4.72 1.89
Langmuir pressure constant (*T* = 380 K; *p* = 50 MPa; *r*_0_ = 3.99 nm)	*P*_L_ =5 × 10^6^ Pa 8 × 10^6^ Pa	13.45 13.43	1.22 1.22	0.41 0.41	1.18 1.18	0.29 0.29	1.88618 1.88608

**Table 12:** Comparison of gas diffusion coefficients in shale from literature and this work.

Reference	Sample	Gas	Temperature (K)	Pressure (MPa)	D (10^−6^ cm^2^·s^−1^)
Du et al. [4]	Modeled kerogen	CO_2_, CH_4_	333	5.0–40	0.4–1.5
Chen. [30]	Changning shale	CO_2_	318	5	0.191
Chen et al. [34]	Longmaxi Shale	CH_4_	355	38	5.62
Kim et al. [35]	Shale matrix (Haenam basin, Korea)	CH_4_	298.15	_	1.82
Zhong et al. [36]	Longmaxi shale	CH_4_	398.15	35	4.577
Wei et al. [43]	SEM-based reconstructed shale digital model	CH_4_ (shale-gas generic model)	300	5	7.56

## Data Availability

The data presented in this study are available on request from the corresponding authors, subject to the approval of the affiliated research institution.

## Supplementary Materials

The following supporting information can be downloaded at: https://www.mdpi.com/article/10.3390/nano16181178/s1.
