# Supplementary material for: Experimental and Theoretical Investigation of Diffusion Characteristics of CH4 and CO2 in Shales
Source: Nanomaterials (Basel). 2026 Sep 17;16(18):1178. doi: 10.3390/nano16181178 (PMC13610075; doi:10.3390/nano16181178)
Supplement: Supplementary file 1 [file nanomaterials-16-01178-s001.zip › nanomaterials-4511651-supplementary.pdf]

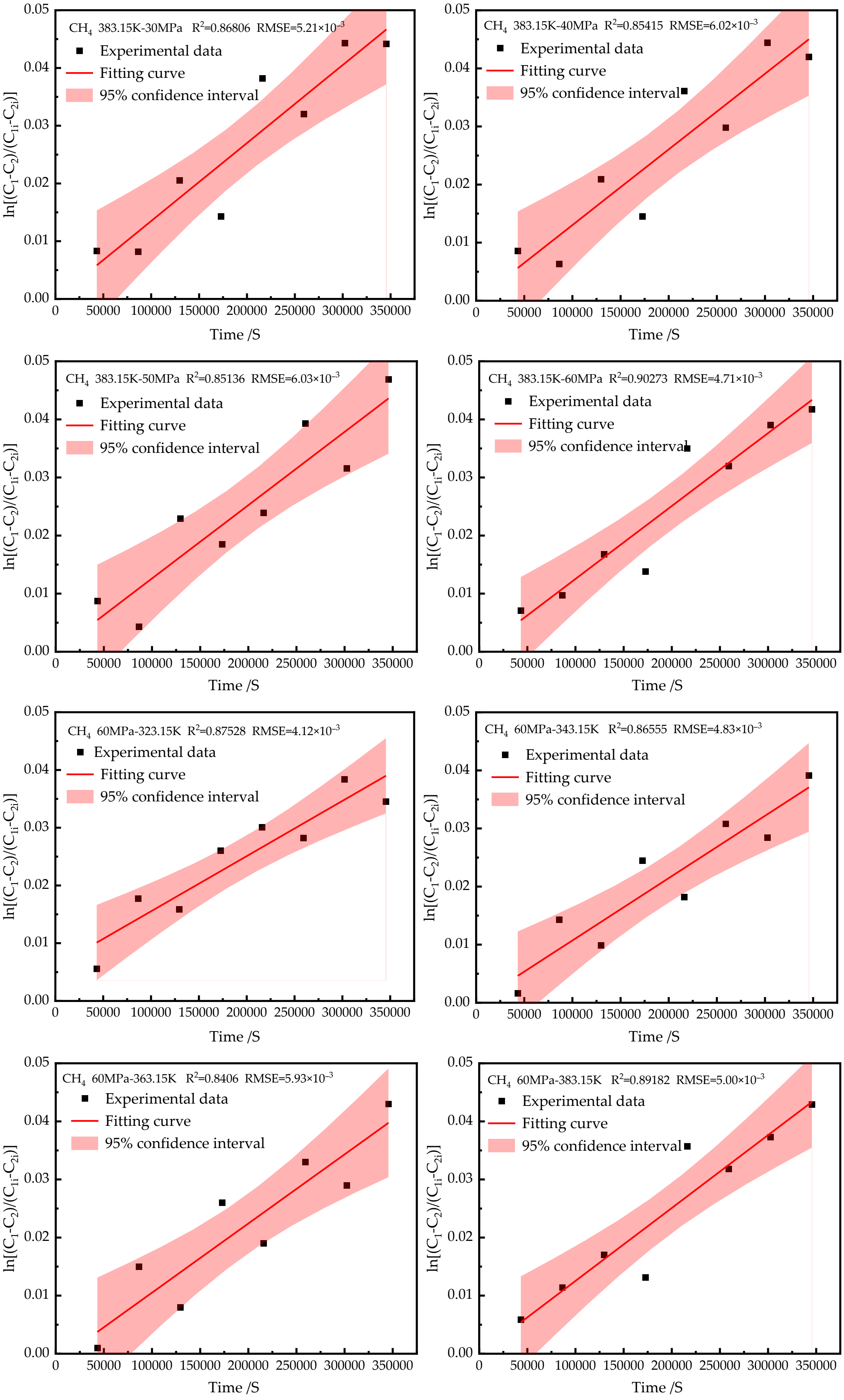

CH4 383.15K-30MPa R2=0.86806 RMSE=5.21×10-3
CH4 383.15K-40MPa R2=0.85415 RMSE=6.02×10-3
CH4 383.15K-50MPa R2=0.85136 RMSE=6.03×10-3
CH4 383.15K-60MPa R2=0.90273 RMSE=4.71×10-3
CH4 60MPa-323.15K R2=0.87528 RMSE=4.12×10-3
CH4 60MPa-343.15K R2=0.86555 RMSE=4.83×10-3
CH4 60MPa-363.15K R2=0.8406 RMSE=5.93×10-3
CH4 60MPa-383.15K R2=0.89182 RMSE=5.00×10-3
Experimental data
Fitting curve
95% confidence interval
ln[(C1-C2)/(C1i-C2i)]
Time /S
0.00
0.01
0.02
0.03
0.04
0.05
0
50000
100000
150000
200000
250000
300000
350000

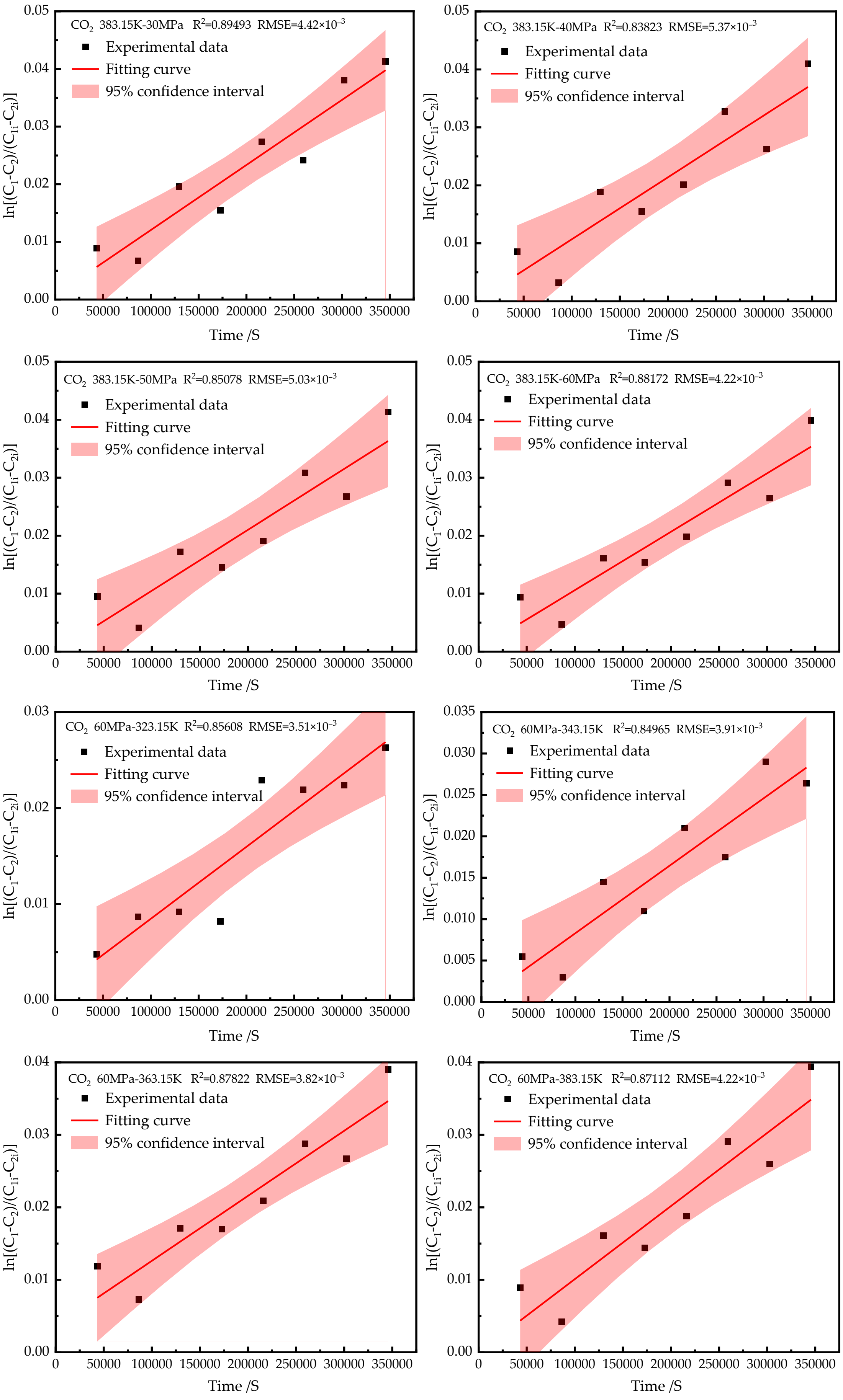

CO2 383.15K-30MPa R2=0.89493 RMSE=4.42×10−3
CO2 383.15K-40MPa R2=0.83823 RMSE=5.37×10−3
CO2 383.15K-50MPa R2=0.85078 RMSE=5.03×10−3
CO2 383.15K-60MPa R2=0.88172 RMSE=4.22×10−3
CO2 60MPa-323.15K R2=0.85608 RMSE=3.51×10−3
CO2 60MPa-343.15K R2=0.84965 RMSE=3.91×10−3
CO2 60MPa-363.15K R2=0.87822 RMSE=3.82×10−3
CO2 60MPa-383.15K R2=0.87112 RMSE=4.22×10−3
Experimental data
Fitting curve
95% confidence interval
ln[(C1-C2)/(C1i-C2i)]
Time /S
0
50000
100000
150000
200000
250000
300000
350000
0.00
0.01
0.02
0.03
0.04
0.05
0.000
0.005
0.010
0.015
0.020
0.025
0.030
0.035
